# Evaluation of the reproducibility of amplicon sequencing with Illumina MiSeq platform

**DOI:** 10.1371/journal.pone.0176716

**Published:** 2017-04-28

**Authors:** Chongqing Wen, Liyou Wu, Yujia Qin, Joy D. Van Nostrand, Daliang Ning, Bo Sun, Kai Xue, Feifei Liu, Ye Deng, Yuting Liang, Jizhong Zhou

**Affiliations:** 1 Institute for Environmental Genomics, Department of Microbiology and Plant Biology, and School of Civil Engineering and Environmental Sciences, University of Oklahoma, Norman, Oklahoma, United States of America; 2 Fisheries College, Guangdong Ocean University, Zhanjiang, Guangdong, China; 3 State Key Laboratory of Soil and Sustainable Agriculture, Institute of Soil Science, Chinese Academy of Sciences, Nanjing, China; 4 CAS Key Laboratory of Environmental Biotechnology, Research Center for Eco-Environmental Sciences, Chinese Academy of Sciences, Beijing, China; 5 State Key Joint Laboratory of Environment Simulation and Pollution Control, School of Environment, Tsinghua University, Beijing, China; 6 Earth Sciences Division, Lawrence Berkeley National Laboratory, Berkeley, California, United States of America; University of Illinois at Chicago, UNITED STATES

## Abstract

Illumina’s MiSeq has become the dominant platform for gene amplicon sequencing in microbial ecology studies; however, various technical concerns, such as reproducibility, still exist. To assess reproducibility, 16S rRNA gene amplicons from 18 soil samples of a reciprocal transplantation experiment were sequenced on an Illumina MiSeq. The V4 region of 16S rRNA gene from each sample was sequenced in triplicate with each replicate having a unique barcode. The average OTU overlap, without considering sequence abundance, at a rarefaction level of 10,323 sequences was 33.4±2.1% and 20.2±1.7% between two and among three technical replicates, respectively. When OTU sequence abundance was considered, the average sequence abundance weighted OTU overlap was 85.6±1.6% and 81.2±2.1% for two and three replicates, respectively. Removing singletons significantly increased the overlap for both (~1–3%, p<0.001). Increasing the sequencing depth to 160,000 reads by deep sequencing increased OTU overlap both when sequence abundance was considered (95%) and when not (44%). However, if singletons were not removed the overlap between two technical replicates (not considering sequence abundance) plateaus at 39% with 30,000 sequences. Diversity measures were not affected by the low overlap as α-diversities were similar among technical replicates while β-diversities (Bray-Curtis) were much smaller among technical replicates than among treatment replicates (e.g., 0.269 vs. 0.374). Higher diversity coverage, but lower OTU overlap, was observed when replicates were sequenced in separate runs. Detrended correspondence analysis indicated that while there was considerable variation among technical replicates, the reproducibility was sufficient for detecting treatment effects for the samples examined. These results suggest that although there is variation among technical replicates, amplicon sequencing on MiSeq is useful for analyzing microbial community structure if used appropriately and with caution. For example, including technical replicates, removing spurious sequences and unrepresentative OTUs, using a clustering method with a high stringency for OTU generation, estimating treatment effects at higher taxonomic levels, and adapting the unique molecular identifier (UMI) and other newly developed methods to lower PCR and sequencing error and to identify true low abundance rare species all can increase reproducibility.

## Introduction

Microorganisms are the most abundant life forms in the biosphere, with an estimated 4–6×10^30^ prokaryotic cells accounting for about 60% of the Earth’s biomass [1]. Microorganisms are highly diverse and play key roles in various ecological processes such as decomposition of organic matter, recycling of essential elements (e.g., carbon, nitrogen, phosphorous, and sulfur) and nutrients, and soil structure formation [2]. However, due to the vast diversity and the uncultivated status of the majority of microorganisms (>99%) in nature [3], detecting, quantifying, and characterizing microbial communities in natural environments is very challenging [4].

Next generation sequencing (NGS) technologies [4], such as 454 pyrosequencing [5], Illumina [6–10], and Ion Torrent [11] platforms, have provided powerful tools to characterize the diversity of microbial communities. However, technical problems inherent in amplicon sequencing have been reported, such as biases in estimation of population abundance in microbial communities [12–14] resulting from PCR primer selection [15–17], PCR template concentration and amplification conditions [18], pooling of multiple barcodes [19, 20], and sequencing itself [18]. In addition, errors introduced by random sampling could lead to an overestimation of microbial community β-diversity [21–25]. These biases are inherent in all sequencing platforms. Pyrosequencing errors have led to an overestimation of the rare biosphere [26]; 16S rRNA amplicon sequencing on the Ion Torrent PGM has been reported to both overestimate and underestimate microbial relative abundance [14]. Results from 16S rRNA amplicon sequencing (2×100 bp paired ends) with an Illumina HiSeq 2000 showed low overlap among replicates [27]. In fact, low overall reproducibility among technical replicates has been frequently reported for various platforms [16, 22, 25, 28–34]. Because of this, it has been argued that technical replicates are required for rigorous interpretation of experimental results [21–23, 28, 35, 36], and that community similarity calculations based on incidence (presence/absence) data may be inaccurate when the number of sequences obtained is insufficient for community representation [16, 23, 34, 37].

Illumina’s MiSeq has become the dominant platform for amplicon sequencing in microbial ecology studies due to its great flexibility, high-throughput, fast-turnaround time, longer sequence reads and higher accuracy [7, 9, 10, 38]. While the increased sampling depth and lower error rate achieved by the Illumina MiSeq may help overcome the inherent biases, it is unclear to what extent technical replicate variation can be reduced. To evaluate the reproducibility of Illumina-based amplicon sequencing, the 16S rRNA gene V4 region of microbial community DNAs from 18 soil samples were amplified in triplicate using unique barcodes for individual replicates as technical replicates. The amplicons were then sequenced on a MiSeq sequencer to examine the technical reproducibility of this platform. Deep sequencing for one soil sample with three technical replicates was also performed to explore how sequencing depth affects reproducibility. Overall, the reproducibility among technical replicates obtained with the MiSeq remains low although it was considerably higher than that for 454 pyrosequencing [22]. Increasing sequencing depth led to higher reproducibility but it plateaued after reaching a certain depth. The results of this study provide guidance for improving amplicon sequencing strategies and experimental design.

## Materials and methods

### Site description and sampling

Soil samples used in this study were collected from a reciprocal transplantation experiment in China designed to simulate climate change. In October 2005, neutral black (B) soil from Hailun (47°26’N, 126°38’E) was transported to Fengqiu (35°00’N, 114°24’E, about 1717 km southwest of Hailun) and Yingtan (28°15N’, 116°55’E, about 2296 km southwest of Hailun) [39–42]. Fengqiu to Yingtan is about 788 km apart. At each location, half of the field site was planted with maize while the other half was not, resulting in six location-treatment combinations: (i) Fengqiu, planted (FP), (ii) Fengqiu, unplanted (FC), (iii) Hailun, planted (HP), (iv) Hailun, unplanted (HC), (v) Yingtan, planted (YP), and (vi) Yingtan, unplanted (YC), each with three field replicates. Surface soil samples (0–20 cm) were collected from experiment plots in Fengqiu, Hailun, and Yingtan in 2011, on September 12, October 2, July 29, respectively, and stored at -80°C until ready for analysis.

### Soil DNA extraction

Soil microbial community DNA was extracted using a freeze-grinding plus sodium dodecyl sulfate (SDS) lysis method as described previously [43] and was purified by gel electrophoresis, followed by phenol extraction. DNA quality was assessed based on the absorbance ratios 260/280 nm and 260/230 nm using a NanoDrop ND-1000 Spectrophotometer (NanoDrop Technologies, Wilmington, DE, USA), and DNA concentration was quantified by PicoGreen (Promega, Sunnyvale, CA, USA) [44] using a FLUOstar Optima plate reader (BMG Labtech, Jena, Germany).

### Sample tagging, PCR library preparation

The primers 515F (5’-GTGCCAGCMGCCGCGGTAA-3’) and 806R (5’-GGACTACHVGGGTWTCTAAT-3’) targeting the V4 hypervariable regions of both bacterial and archaeal 16S rRNA genes were used. Both forward and reverse primers contained Illumina adapter, pad, and linker sequences. The reverse primers also contained a barcode sequence (12-mer) between the Illumina adapter and pad sequences to allow pooling of multiple samples in one sequencing run [7]. All primers were synthesized by Life Technologies (Carlsbad, CA, USA) (S1 Table).

Three libraries with unique tags were generated for each soil sample as technical replicates (S1 Table). Each amplification reaction had a total volume of 25 μl containing 2.5 μl 10×PCR buffer II (including dNTPs), 0.5 unit of AccuPrime^™^ Taq DNA Polymerase High Fidelity (Life Technologies), 0.4 μM of each primer, and 10 ng template soil DNA. Reactions were carried out on a Gene Amp PCR-System^®^ 9700 (Applied Biosystems, Foster City, CA, USA). Thermal cycling conditions were as follows: an initial denaturation at 94°C for 1 min, and 30 cycles at 94°C for 20 s, 53°C for 25 s, and 68°C for 45 s, with a final extension at 68°C for 10 min.

Following amplification, 2 μl of PCR product from each reaction was used for agarose gel (1%) electrophoresis to confirm amplification. Each library was generated by pooling the triplicate PCR reactions and quantifying with PicoGreen. A 200 ng aliquot of PCR product from each library was then pooled for one MiSeq sequencing run. The pooled mixture was purified using a QIAquick Gel Extraction Kit (QIAGEN Sciences, Germantown, MD, USA) and analyzed on an Agilent 2100 Bioanalyzer with a High Sensitivity DNA Chip (Agilent Technologies, Waldbronn, Germany) for size confirmation, and then re-quantified with PicoGreen.

### Sequencing

Sample libraries for sequencing were prepared per the MiSeq Reagent Kit Preparation Guide (Illumina, San Diego, CA, USA) as described previously (Caporaso et al 2012). Briefly, the combined sample library was diluted to 2 nM, denatured with 0.2 N fresh NaOH, diluted to 8 pM by addition of Illumina HT1 buffer, and then mixed with an equal volume of 8 pM PhiX (Illumina, San Diego, CA, USA). The library (600 μl) was loaded with read 1, read 2 and index sequencing primers [7] on a 300-cycle (2×150 paired ends) reagent cartridge (Illumina), and run on a MiSeq sequencer (Illumina).

Two independent experiments were designed to compare variations among technical replicates when replicates are sequenced in the same run (Experiment I) or when sequenced in three separate runs (Experiment II) (S2 Table). For Experiment I, all libraries of the 18 soil samples, each having three unique tagged libraries, were pooled (54 libraries total) with another 16 libraries from unrelated experiments and sequenced in one MiSeq run (70 libraries total). For Experiment II, the three unique tagged libraries of each sample were arranged into three independent library pools (18 libraries per pool) and sequenced in three separate MiSeq runs. Each of these three pools was combined with 75–77 other libraries from unrelated experiments (93 to 95 libraries total for each run). The cluster densities for all runs were in the range of 453–580 k/mm^2^, and had 93–95% of clusters passing filters, 80–85% of bases with Q≥30; and 46–54% of reads aligned to Phix (S2 Table). An extra deep sequencing was performed on a 500 cycle (2x250 paired ends) reagent cartridge (Illumina) for one triplicate set of barcoded libraries from one of the soil samples along with 210 unrelated libraries.

### Sequence data processing

Raw sequence data was processed using an in-house pipeline which was built on the Galaxy platform and incorporated various software tools. First, the quality of the raw sequence data was evaluated with FastQC (http://www.bioinformatics.babraham.ac.uk/projects/fastqc/). Then, demultiplexing was performed to remove PhiX sequences, discard sequences without barcodes, and sort the sequences into the appropriate tagged libraries based on their barcodes. To minimize sequencing errors and ensure sequence quality, both forward and reverse reads were trimmed based on the sequence quality score using Btrim [45]. Sequences were trimmed if the average quality score of 5 continuous bases was less than 20. Sequences less than 30 bases or contained undetermined bases, ‘N’, were removed. Paired end reads with sufficient overlap (minimum 20 base overlap between forward and reverse reads) were merged into full length sequences by FLASH v1.2.5 [46]. Reads that could not be joined were removed. These steps were followed to avoid issues with over inflation of sequencing error rates by FLASH. Chimeric sequences were discarded based on predictions by Uchime (usearch v5.2.3) [47] using the Greengenes database [48] for 16S rRNA gene sequences as a reference. OTUs were clustered using Uclust (usearch v5.2.32) [49] at a 97% similarity level by a de novo picking method. For the deep sequencing run, OTUs were clustered using both Uclust and UPARSE [50] with de novo OTU picking method. Final OTUs were generated based on the clustering results, and taxonomic annotation of individual OTUs was based on representative sequences using RDP’s 16S Classifier 2.5 and the associated training set published with this version [51]. To control variation resulting from an unequal number of sequences across samples, sequence resampling was performed for each sample. Sequence resampling was performed after OTU generation at a rarefication sequence level based on the sample with the fewest number of sequences. The re-sampling procedure was done using a Perl script developed in our lab. Sequences from each sample are randomly drawn from the original pool until the rarefication sequence level is achieved. Once a sequence is drawn, it is excluded from further rounds of selection to prevent repetition. Where Singletons, defined as OTUs with only one sequence and presenting in only one technical replicate across all samples, were removed, they were done so prior to resampling. The effect of unique OTUs, defined as OTUs with one or more sequences and presenting in only one technical replicate across all samples, on reproducibility was determined by comparing the reproducibility between OTU sets containing these unique OTUs and sets where unique OTUs were removed after resequencing.

### Statistical analysis

Microsoft Excel was used to calculate OTU overlap and sequence abundance weighted OTU overlap (weighted OTU overlap, here on out) between/among technical replicates using the formulas below:

1. OTU overlap between two technical replicates = 2 x shared OTUs / (OTUs of replicate A + OTUs of replicate B);
2. OTU overlap among three technical replicates = 3 x shared OTUs / (OTUs of replicate A + OTUs of replicate B + OTUs of replicate C);
3. Weighted OTU overlap between two technical replicates = (the number of sequences within the shared OTUs of replicate A + the number of sequences within the shared OTUs of replicate B) / the total number of sequences within both replicates A and B;
4. Weighted OTU overlap among three technical replicates = (the number of sequences within the shared OTUs of replicate A + the number of sequences within the shared OTUs of replicate B + the number of sequences within the shared OTUs of replicate C) / the total number of sequences from replicates A, B, and C.

Rarefaction analysis based on both treatment and technical replicates was done after sequence re-sampling using the Mothur program (Patrick Schloss, http://www.mothur.org/) [52]. Maximum OTUs were predicted at different levels based on individual tags and treatments using the Chao1 method [53]. Shannon–Weaver index (H’) [54], Pielou evenness index (J) [55], and number of OTUs were used to measure the microbial α-diversity and evenness for each library. Three-way ANOVA was used to test the differences of Shannon–Weaver index (H’), Pielou evenness index (J), and number of OTUs among technical replicates, biological replicates, treatments and experiment locations.

Both Sorensen similarity (S_s_) and Bray-Curtis similarity (BC_s_) were calculated between any pair of tagged PCR libraries. The complement of Sorensen similarity (S_d_ = 1—S_s_) and the complement of Bray-Curtis similarity (BC_d_ = 1—BC_s_) were used to measure the β-diversity of microbial communities between any two tagged PCR libraries. One-way analysis of variance (ANOVA) was used to compare β-diversities among technical replicates, treatments, and experiment locations. The Duncan multiple range test [56] was used to determine the statistical significance of the observed differences in β-diversity. To confirm that the differences among technical replicates were not just random variation, PERMDISP (permutation test of multivariate homogeneity of groups dispersions) analysis [57, 58] based on a null model was performed against the null hypothesis that the three technical replicates of each soil sample came from the same underlying microbial community for all 18 samples of experiment I. The null model algorithm randomly draws individuals from all OTUs with probabilities proportional to the average relative abundance of the OTUs in all technical replicates of the same sample. By this null model, we generated 3 null replicates for each sample, compared their dispersions with that of the observed technical replicates by permutational test, and repeated this procedure 1000 times.

Detrended correspondence analysis (DCA) [59] was used to determine the overall phylogenetic compositional differences of microbial communities using CANOCO 4.5 (Biometris—Plant Research International, Wageningen, The Netherlands).

## Results

### Overview of sequencing data statistics

Technical replicates of each soil sample were sequenced in either the same (Experiment I) or three different (Experiment II) sequencing runs. In Experiment I (18 soils, 3 technical replicates per soil, all replicates in one run), an average of 16,751±2,188 raw sequence reads (paired ends) were obtained with an average effective combined sequence number of 15,349±1,862 (about 8.25% of reads were lost after quality trimming and combining) and an average of 3,060±398 OTUs (97% sequence similarity) per library (S3 Table). In Experiment II (18 soils, 3 technical replicates per soil, 3 runs, one replicate per run), an average of 10,072±3,174 paired end reads were obtained with an average effective combined sequence number of 9,252±2,912 (about 8.02% of reads were lost after quality trimming and combining) and an average of 2,191±508 OTUs (97% sequence similarity) per library (S4 Table).

Rarefaction analyses and Chao1 estimation were performed at both the treatment and technical replicate levels. For Experiment I, an average of 10,035±1,007 OTUs were observed at the treatment level, with an average OTU coverage of 0.55±0.03 (S1A Fig) based on Chao 1 estimation, and an average of 30,60±398 OTUs were observed at the technical replicate level, with an average OTU coverage of 0.48±0.03 (S3 Table). For Experiment II, an average of 6,881±548 OTUs were observed at the treatment level (average OTU coverages of 0.61±0.03, S1B Fig), and an average of 2,191±508 OTUs at the technical replicate level (average OTU coverage, 0.5±0.04, S4 Table). Although fewer sequences and OTUs were obtained when technical replicates were sequenced separately, the OTU coverage was relatively higher at both the treatment and technical replicate levels (treatment level, p<0.0001; technical replicate level, p = 0.002). But, based on the OTU coverage of both experiments, the diversity of the abundant populations in these communities was recovered reasonably well [60] in this study.

### OTU overlap among technical replicates

**Fig 1:**
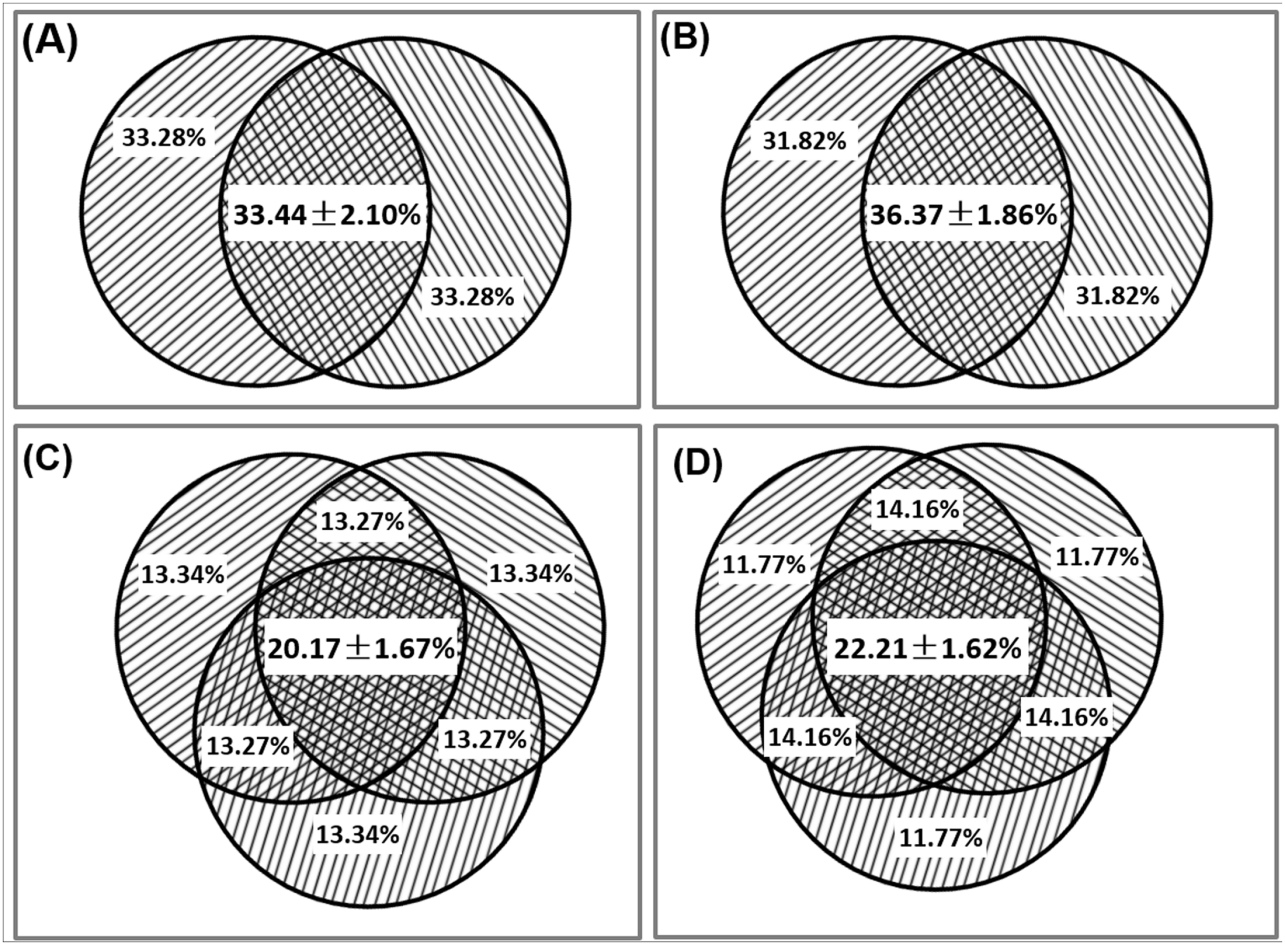
OTU overlap among technical replicates for experiment I. (A) Between two technical replicates, singletons not removed; (B) between two technical replicates, singletons removed; (C) among three technical replicates, singletons not removed; (D) among three technical replicates, singletons removed.

If all populations in a community were sampled, we would expect 100% reproducibility in the OTUs detected for technical replicates. However, in practice, this is difficult to achieve due to under-sampling, random-sampling, artifacts, and/or the complexity of the microbial communities [4, 21–23]. To determine the reproducibility of the MiSeq platform, OTU overlap was determined for the sequenced technical replicates with and without singleton OTUs. For experiment I, without removing singletons, the average OTU overlap (presence/absence) between two technical replicates, when the sequences of each sample were resampled at a rarefication level of 10,323 sequences, was 33.4±2.1% (Fig 1A; S5 Table) and 20.2±1.7% among three replicates (Fig 1C; S5 Table). OTU overlap was lower for Experiment II (p<0.001 for both two and three replicates), with an average overlap of 30.6±1.6% (S2A Fig; S6 Table) between two technical replicates and 17.4±1.1% among three replicates (S2C Fig; S6 Table), when the sequences of each sample were resampled at a rarefication level of 4130 sequences.

The OTU overlap increased when singleton sequences were removed. For Experiment I, the OTU overlap among three technical replicates was 22.2±1.6% (S5 Table; Fig 1D, *p*<0.001) and 18.3±1.2% (S6 Table; S2D Fig; p<0.001) for Experiment II. As observed in the previous comparisons, the OTU overlap between two technical replicates was higher than for three replicates.

Weighted OTU overlap was also calculated. For Experiment I, the average weighted OTU overlap among three technical replicates at a rarefication level of 10,323 sequences was 81.2±2.1% (S7 Table). The weighted overlaps were significantly lower for Experiment II (p<0.0001), with an average of 71.5±3.9% among three replicates at a rarefication level of 4130 sequences (S8 Table). Like 454 pyrosequencing [22], the variation in amplicon sequencing of technical replicates on the MiSeq is fairly high when sequence abundance is not considered, but is much lower if sequence abundance is considered. As observed with overlap based on presence/absence, removal of singletons also increased the weighted OTU overlap (*p*<0.001, S7 and S8 Tables).

Although singleton OTUs were removed before sequence resampling, unique OTUs were detected after resampling. These unique OTUs were also removed based on the assumption that they could be sequencing artifacts. When the unique OTUs having only one sequence were removed, OTU overlap increased by about 1 percent. Removal of the unique OTUs having two or more sequences did not increase overlap further (S9 Table).

### Sequencing depth vs OTU overlap

**Fig 2:**
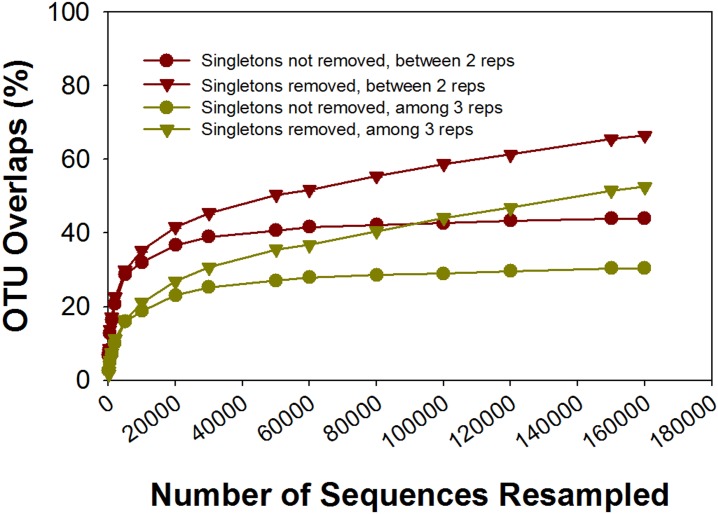
Overlap of OTUs generated using Uclust between/among technical replicates at different sequencing depth. At a sequencing depth of about 30,000 reads, overlap of both two and three replicates was approaching a plateau when singletons were not removed.

A greater sampling effort would be expected to increase overlap among technical replicates. To test this, a set of technical replicates from one sample was deep sequenced. Over 160,000 sequences were obtained for each of the three technical replicates. Sequences from each technical replicate were randomly resampled at different depths (100 to 160,000) and then the OTU overlap was calculated at each depth. The data described in this section is for the average OTU overlap between every two of the three technical replicates with singletons present; data for other comparisons is shown in Fig 2 and S3 Fig, and S10 and S11 Tables. OTU overlap increased from 20.7±0.1% for 2000 sequences to 43.9±0.7% for 160,000 (S10 Table). Weighted OTU overlap increased from 56.7±0.5% to 95.3±0.1% (S11 Table). At a sequencing depth of about 30,000 reads, OTU overlap reached a plateau at 39.0% (Fig 2; S10 Table). Weighted OTU overlap also approached saturation at a depth of 30,000 sequences, but plateaued at 90.4% (S3 Fig; S11 Table).

UPARSE was used for OTU generation in the deep sequencing experiment because it is believed to reduce spurious OTUs, minimize the effect of sequencing errors, reduce OTU inflation, and more closely reflect the true community diversity [50]. While the effective sequence number (120,000 per technical replicate) and the total number of OTUs decreased by 66.4% (total OTU: 24,745, Uclust; 8,309, UPARSE) when UPARSE was used, the OTU overlap increased. For example, if singletons were not removed, the OTU overlap between two technical replicates was 50.1% at a sequencing depth of 30,000 reads (S12 Table, S4 Fig), about 10% higher than with Uclust, and was as high as 57.9% at a sequencing depth of 120,000 reads. However, the saturation point remained at 30,000 sequences (S12 Table, S4 Fig). The results indicate that using UPARSE improved the estimation of reproducibility considerably.

### Effect of technical variation on diversity estimation and differentiation of microbial communities

To determine how variation among technical replicates affects the estimation of microbial local diversity (α-diversity), the Shannon-Weaver index, number of OTUs, and Pielou evenness were calculated for technical replicates, treatments, and experiment locations. Three-way ANOVA indicated that all diversity measures were significantly different among treatment regardless of whether the replicates were run in separate runs or the same one (Table 1; S13 Table). When the replicates were sequenced in separate runs, experimental locations were also significantly different by all measures (S13 Table). When the replicates were sequenced in the same run, only the number of OTUs was significant for experiment location (Table 1). No significant difference was detected among technical replicates regardless of whether they were sequenced in separate runs or in the same run. These results suggest that the variation in technical replicates may not affect the estimation of α-diversity for treatment effects and differences among experimental locations, but sequencing technical replicates in separate runs increased species coverage and may make the detection of community differences easier.

**Table 1:** Three-way ANOVA ^a^ to assess alpha diversities ^b^ at different levels for experiment I.

	Df	Shannon measurement (H')				Number of OTUs				Pielou evenness (J)			
		With singletons		Singletons removed		With singletons		Singletons removed		With singletons		Singletons removed	
		F ^c^	P ^d^	F	P	F	P	F	P	F	P	F	P
**Technical replicate**	2	0.028	0.973	0.014	0.986	0.232	0.794	0.132	0.877	0.017	0.983	0.005	0.995
**Location**	2	1.913	0.159	1.470	0.240	13.976	1.7E-05**	11.273	9.7E-05**	0.698	0.502	1.439	0.247
**Treatment**	1	14.421	4.1E-04**	13.665	0.001**	14.164	4.6E-04**	10.889	0.002**	14.081	4.7E-04**	18.907	7.1E-05**
**Residuals**	48												

^a^ The model used for the three-way ANOVA: (α diversity)_ijk_ = (technical replicate)_i_+(location)_j_+(treatment)_k_+ (error)_ijk_.

^b^ The Shannon entropy index, number of OTUs, and the Pielou evenness were used to measure the alpha diversity for each tagged PCR library.

^c^ F-value

^d^ P-value (>F)

* p<0.05

** p<0.01

To understand how technical variation affects the comparison of different microbial communities (i.e., β-diversity), two popular dissimilarity metrics, Sorensen’s incidence-based and Bray-Curtis’s abundance-based dissimilarities were calculated using combined OTU data. These metrics are widely used in many studies and range from 0 to 1, with 0 indicating that all OTUs/individuals are shared between two communities while 1 indicates no OTUs/individuals are shared. Results from both methods showed the same trend although β-diversity was always lower with Bray-Curtis. β-diversity increased in the order of technical replicates < biological replicates < treatments within location < planted across locations < unplanted across locations based on comparisons by Duncan grouping (Table 2). Removing singletons did not significantly change the β-diversities at any level. Similar results were obtained from sequencing the replicates in three separate runs (S14 Table). No significant difference was observed between β-diversities for the technical replicates sequenced in the same run or in separate runs. The PERMDISP analysis showed that the dispersions of the observed technical replicates were always significantly larger than those of null replicates (>12%, P = 0.001, S15 Table), suggesting that the differences between technical replicates were significantly larger than random variation. In other words, the differences between technical replicates could not be simply caused by random sequence sampling. In addition, the unweighted dissimilarity index (Sorensen) always resulted in larger F values and relative differences (Δd%) between observed and null dispersions compared to the abundance-weighted index (Bray-Curtis), indicating that biases other than random sampling had less of an effect on abundance-weighted metrics (S15 Table).

**Table 2:** One-way ANOVA and Duncan grouping to assess β-diversity at different levels based on OTUs from Experiment I.

	Data size ^a^	With singletons				Singletons removed			
		Sorensen		Bray-Curtis		Sorensen		Bray-Curtis	
		β diversity ^b^	Significance ^c^	β diversity ^b^	Significance ^c^	β diversity ^b^	Significance ^c^	β diversity ^b^	Significance ^c^
**At technical replicate level**	54	0.499	e	0.269	d	0.466	e	0.258	d
**At biological replicate level**	162	0.528	d	0.374	c	0.497	d	0.364	c
**Between treatments (Planted/ unplanted) within locations**	243	0.561	c	0.422	b	0.531	c	0.413	b
**Among planted across locations**	243	0.617	b	0.505	a	0.594	b	0.500	a
**Among unplanted across locations**	243	0.637	a	0.523	a	0.609	a	0.514	a

^a^ Data sizes (n) are the number of data points of the pairwise comparisons within the technical replicates, biological replicates, or treatments.

^b^ We calculated two popular β-diversity dissimilarity measurements, Sorensen and Bray-Curtis, in which Sorensen dissimilarity is based on OTUs richness and Bray-Curtis dissimilarity takes OTUs abundance into account.

^c^ Significance at [pr(>F)] <0.05, using the Duncan grouping method. a, b, c, d and e represent the significance of the β-diversity differences between technical replicates, biological replicates, and the treatments. The letter ‘a’ indicates the two highest β-diversities, although the second highest diversity is not significant, those diversities that are significantly lower than the highest diversity are indicated by the letter b, c, d, or e.

**Fig 3:**
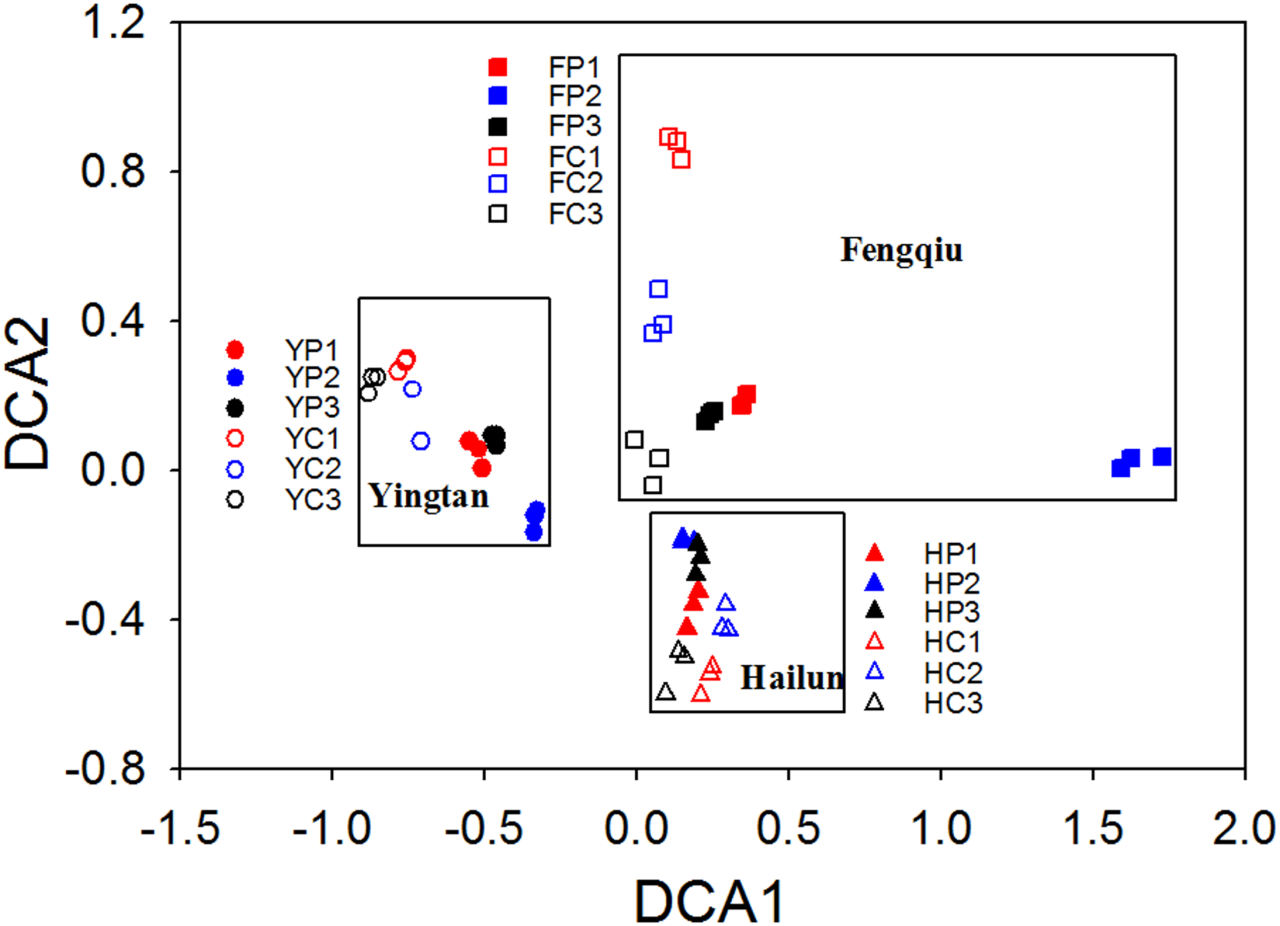
Detrended correspondance analysis of the microbial communities from Experiment I, with singletons not removed. Samples were from three experiment locations (H, Hailun; F, Fengqiu; Y, Yingtan) with two treatments at each location: planted (P) and unplanted (C, control), and three field replicates for each treatment. Each soil was tagged three times to create technical replicates. The three technical replicates of each soil were sequenced in the same MiSeq run. Singletons were not removed.

To understand whether technical variation affects the differentiation of microbial communities under different treatments or at different experiment locations in this study, DCA was performed using the OTU data. The DCA plot shows that technical replicates clustered together tightly. The communities separated first by location (Hailun, Fengqiu, and Yingtan), then by treatment (planted or unplanted), and finally by biological replicates (Fig 3). Similar groupings were obtained with and without singletons and whether the technical replicates were sequenced together or in separate runs (S5A–S5C Fig). These results indicate that the largest variation in composition and structure of the communities came from differences in location and treatment for these samples. The community variation among the biological replicates was also obviously larger than that for technical replicates.

## Discussion

Recent developments and applications in metagenomics methods such as high-throughput sequencing [61–63], especially targeted gene amplicon sequencing [6–9], have enabled rapid acquisition of microbial community structure and composition information at community-wide scales. This has allowed scientists to rapidly analyze microbial communities and address interesting hypotheses in microbial biodiversity and biogeography [64]. However, there are concerns in using high-throughput sequencing data to generate and test these hypotheses [64] due to bias, artifacts, and variations [15, 26]. We have previously shown that there is considerable variation in 454 pyrosequencing results related to microbial community composition and structure between/among technical replicates due to random sampling and low sampling effort [21, 22].

Target gene amplicon sequencing with the MiSeq has been evaluated in terms of technology validation [8, 38, 65], appropriate protocols [6, 10, 66], data analysis methods [38, 50], analyses of error [8, 67], bias [18, 37, 65], artifacts [38], ecological inference [37], and comparison to other platforms, primarily 454 pyrosequencing [37, 65, 67]. We report here a systematic analysis of the reproducibility of MiSeq amplicon sequencing. Based on our analysis, bias due to random and low effort sampling and overestimation of β-diversity is still an issue with MiSeq amplicon sequencing, as previously reported for 454 pyrosequencing [22], although the level of bias is greatly improved with increased sampling effort. We compared the reproducibility of 16S rRNA gene amplicon sequencing on the MiSeq to that previously reported for 454 pyrosequencing [22]. At about a 5 times greater sequencing depth (~10,000 sequences per sample, MiSeq sequencing; ~2,000 sequences per sample, 454 pyrosequencing), OTU overlap with MiSeq was about 20% greater than with 454 sequencing (33.4±2.1%, MiSeq sequencing; 13.1±1.5%, 454 pyrosequencing) between two technical replicates (not considering sequence abundance), and over 10% greater among three technical replicates (20.2±1.7%, MiSeq sequencing; 5.9±1.6%, 454 pyrosequencing). In addition, the PERMDISP analysis showed that the variation of observed technical replicates was always larger than that of null replicates, indicating there were real differences among technical replicates. This confirms that random and low effort sampling could lead to overestimation of microbial β-diversity in microbial target gene amplicon sequencing analysis, and make comparison across samples difficult [21, 22]. Our deep sequencing results also confirm that increasing sampling effort increases reproducibility of amplicon sequencing and reduces the variation among technical replicates. When compared at the same sequence depth (2,000 sequences), the OTU overlap between two technical replicates of Miseq sequencing was still higher than that of 454 pyrosequencing (20.7±1.0% for MiSeq, 13.1±1.5% for 454 pyrosequencing [22]). This could be because Illumina sequencing has a lower sequencing error rate [68] which reduced the proportion of spurious OTUs in each technical replicate.

One critical question is whether the observed variation among technical replicates can be fully overcome by deep sequencing, and if so, how deep must the sequencing depth be. Our results suggest that merely increasing sequencing depth does not entirely overcome the variation among technical replicates. The OTU overlap did increase with sequencing depth, but plateaued at about 30,000 sequences, the saturation point, when Uclust was used. Removing singletons likely also removed artifacts, thereby increasing the saturation point to 150,000 sequences with a higher plateau. However, even with the removal of suspect OTUs, the plateau (between two technical replicates) was still only 65.5%. When UPARSE was used to generate OTUs, a higher saturation point, 60,000 effective sequences, was observed, with a higher plateau. Removing singletons further improved the UPARSE OTU overlap, with an estimated saturation point of 130,000 effective sequences, and a plateau of about 80%. As was discussed previously for 454 sequencing [21, 22], in addition to sequencing, there are multiple steps subject to random sampling effects, affecting the reproducibility of MiSeq amplicon sequencing, such as field sampling, DNA extraction, PCR amplification, sample pooling, and sample loading for sequencing. While increasing the sequence depth is one way of increasing sampling effort, it is not the only factor impacting reproducibility. Sampling effort can also be increased at earlier steps of sample preparation, such as by composite sampling from multiple field-sampling points, increasing sample scale for DNA extraction, combining multiple DNA extracts, increasing the amount of DNA template in PCR reactions and performing multiple PCR reactions. As suggested by our results, for 16S rRNA gene amplicon sequencing on the MiSeq, 30,000 effective sequences should be considered a minimum requirement for the analysis of soil microbial communities if other conditions are left unchanged. Other environments may require different minimums based on how complex and diverse the microbial communities are in those environments.

Using either method, Bray-Curtis or Sorensen, the estimated β-diversity among technical replicates was significantly smaller than among treatment and biological replicates. However, the β-diversity among technical replicates was only about 20% less using the Sorensen method, while it was about 40% less with Bray-Curtis. This suggests that the Bray-Curtis β-diversity is a more sensitive measure for detecting treatment effects. For dominant OTUs, variation among technical replicates affects the abundances of these OTUs and is primarily due to random sampling. For rare OTUs, the variation is likely from both random sampling and sequencing artifacts, including chimeras and sequencing errors. Although in this study, the sequence variation among technical replicates did not affect common α-diversity indices, caution should be taken in interpreting the microbial community composition due to possible artifacts.

Removing singletons, which may account for a large proportion of unrepresentative OTUs, significantly increased OTU overlap between/among technical replicates for both general and deep sequencing. Some previous studies have found that most pyrosequencing singletons were artifacts [26, 69, 70]; however, other studies have found that some of these singleton OTUs had high similarity to known sequences, suggesting that some singletons may actually reflect rare lineages/genotypes in the community [25, 71]. As such, it is important to refine how singletons are defined to include OTUs that may be part of the rare biosphere, while still minimizing the risk of including artifacts. One possible solution is to remove those OTUs containing only a single sequence and present in only one technical replicate of an experiment, because OTUs detected in only a single technical replicate are more likely to be artifacts, even if the OTU contains two or more sequences. However, removing unique OTUs with two or more sequences is likely to have only a minimal effect on overlap, as these were rarely detected. Indexing individual template molecules with a unique molecular identifier (UMI) before PCR and deep sequencing could be a promising method for detecting low frequency true rare species, as true rare species could be distinguished from PCR errors or sequencing errors based on consensus among reads sharing the same index [72–75]; however, this method need more test prior to it can be used routinely based on our own experiments (not shown).

OTU overlap increased by more than 10% when OTUs were generated using UPARSE compared to Uclust due to removal of more rare OTUs [50]. Nevertheless, UPARSE resulted in a 30% reduction of effective sequences and a 66% reduction of OTUs. OTU reduction using UPARSE was also reported by other researchers [69, 76] likely due to the greater restrictions against chimeras and other artifacts [50]. This suggests that while true artifacts are being removed, some rare species may be discarded as well. OTUs generated by UPARSE may be more precise [69], however, α- and β-diversity measurements based on OTU data generated by both Uclust and UPARSE were concordant [76].

The reproducibility of MiSeq amplicon sequencing was compared within (Experiment I) and across runs (Experiment II). Experiment II had less OTU overlap than Experiment I. Removing singletons did increase OTU overlap for all experiments, although to a lesser degree in Experiment II. These results indicate that sequence data among technical replicates has a greater variation and is less reproducible when the replicates are sequenced in separate runs than when sequenced in the same run. This could likely be due to both slight procedural and reagent differences during sample preparation. This could also be the reason that experiment II showed significantly higher diversity coverage at both treatment and technical levels and a higher sensitivity for detecting differences in microbial community α-diversity among experiment locations. Regardless, these results suggest that all samples from an experiment should be sequenced in the same run to avoid additional variation from sequencing that may obscure real treatment/site differences. This is consistent with what was reported for 454 pyrosequencing [34]. If there are too many samples from an experiment to be sequenced within one run, treatment replicates (biological or technical) should be split evenly into multiple runs.

MiSeq amplicon sequencing of target genes is rapidly becoming a leading method for profiling microbial communities. While it provides a larger sequence data output and a greater sampling effort, MiSeq sequencing still suffers from similar problems as 454 pyrosequencing, including variation in technical replicates and low reproducibility due to random and low effort sampling [21, 22]. This study highlighted several strategies that can be used to overcome some of these issues. For example, increasing sequencing depth (to an optimal depth for a given environment), removing singletons and other unrepresentative OTUs, and using UPARSE to generate OTUs does remove some of the variation in technical replicates. Other methods, such as high stringency quality trimming and chimera removal are helpful as well. Consistent with previous studies the application of the amplicon sequencing on the MiSeq to analyze microbial community from a reciprocal transplantation experiment simulating climate change in China [39–42] revealed significant differences between planted and un-planted plots, and among different experiment locations. These results suggest that amplicon sequencing on the MiSeq is useful for analyzing microbial community structure if used appropriately and with caution. For example, by performing technical replicates, sequencing all samples from one experiment in a single run or evenly splitting biological replicates and/or technical replicates from each treatment into multiple runs, removing spurious sequences and unrepresentative OTUs, using a clustering method with high stringency for OTU generation, estimating treatment effects at higher taxonomic levels, and adapting UMI and other newly developed methods to lower PCR and sequence errors and to identify true low abundance rare species.

## Supporting information

S1 Fig A and B rarefaction analysis based on treatments for experiment I (A) and experiment II (B). (PDF)

S2 Fig OTU overlap between/among technical replicates for experiment II. (A) Between two technical replicates, singletons not removed; (B) between two technical replicates, singletons removed; (C) among three technical replicates, singletons not removed; (D) among three technical replicates, singletons removed.
(PDF)

S3 Fig Overlap of OTUs generated with sequence abundance using Uclust between/among technical replicates at different sequencing depth. At a sequencing depth of about 30,000 reads, overlap of both two and three replicates were reaching a plateau no matter singletons were removed or not.
(PDF)

S4 Fig Overlap of OTUs generated using UPARSE between/among technical replicates at different sequencing depth. At a sequencing depth of about 30,000 reads, overlap of both two and three replicates were reaching a plateau when singletons were not removed.
(PDF)

S5 Fig S5A-C Figs Detrented corresponding analysis of the microbial communities for experiment I, with singletons removed (A), experiment II, with singletons not removed (B), experiment II, with singletons removed (C). Samples were from three experiment locations (H, Hailun; F, Fengqiu; Y, Yingtan) with two treatments at each location: planted (P) and unplanted (C, control), each treatment with three field replicates. Each soil was tagged three times as technical replicates. For experiment I, the three technical replicates of each soil were sequenced in the same MiSeq run, and for experiment II, the three technical replicates of each soil were sequenced in three different MiSeq runs.
(PDF)

S1 Table S1A-C Table. Sequencing primers and PCR forward primers (A), PCR reverse primers for experiment I (B), PCR reverse primers for experiment II (C). (XLS)

S2 Table Sample (tagged PCR libraries) arrangement in Miseq runs and sequencing parameters. (PDF)

S3 Table Summary of sequencing statistics for experiment I. (PDF)

S4 Table Summary of sequencing statistics for experiment II. (PDF)

S5 Table OTU overlap between/among technical replicates for experiment I. (PDF)

S6 Table OTU overlap between/among technical replicates for experiment II. (PDF)

S7 Table Sequence abundance weighted OTU overlap between/among technical replicates for experiment I. (PDF)

S8 Table Sequence abundance weighted OTU overlap between/among technical replicates for experiment II. (PDF)

S9 Table Effect of removing unique OTUs after sequence resampling on OTU overlap between/among technical replicates. (PDF)

S10 Table OTU overlap between/among technical replicates at different sequencing depth. (PDF)

S11 Table Sequence abundance weighted OTU overlap between/among technical replicates at different sequence depth. (PDF)

S12 Table OTU overlap between/among technical replicates at different sequencing depth with OTUs generated by UPARSE. (PDF)

S13 Table Three-way ANOVA to assess alpha diversities b at different levels for experiment II. (PDF)

S14 Table One-way ANOVA and Duncan grouping to assess β-diversity at different levels based on OTUs for experiment II. (PDF)

S15 Table Comparison of dispersions between observed technical replicates and those between null replicatesa by permutation test of multivariate homogeneity of groups dispersions (PERMDISP). (PDF)
