# Supplementary material for: Evaluation of the reproducibility of amplicon sequencing with Illumina MiSeq platform
Source: PLoS One. 2017 Apr 28;12(4):e0176716. doi: 10.1371/journal.pone.0176716 (PMC5409056; doi:10.1371/journal.pone.0176716)
Supplement: S1 Fig — (PDF) [file pone.0176716.s001.pdf]

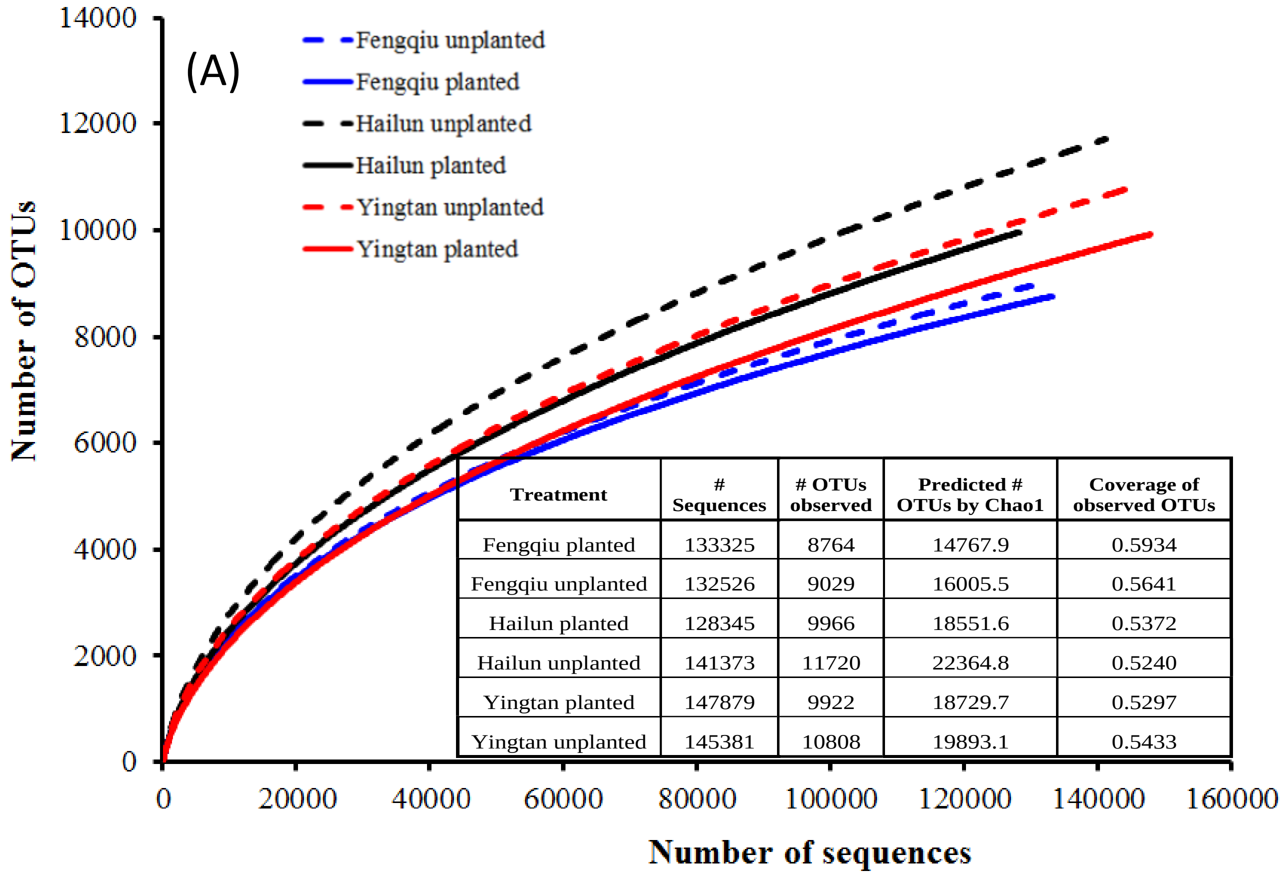

Treatment	# Sequences	# OTUs observed	Predicted # OTUs by Chao1	Coverage of observed OTUs
Fengqiu planted	133325	8764	14767.9	0.5934
Fengqiu unplanted	132526	9029	16005.5	0.5641
Hailun planted	128345	9966	18551.6	0.5372
Hailun unplanted	141373	11720	22364.8	0.5240
Yingtan planted	147879	9922	18729.7	0.5297
Yingtan unplanted	145381	10808	19893.1	0.5433

S1A Fig. Rarefaction analysis based on treatments for experiment I.

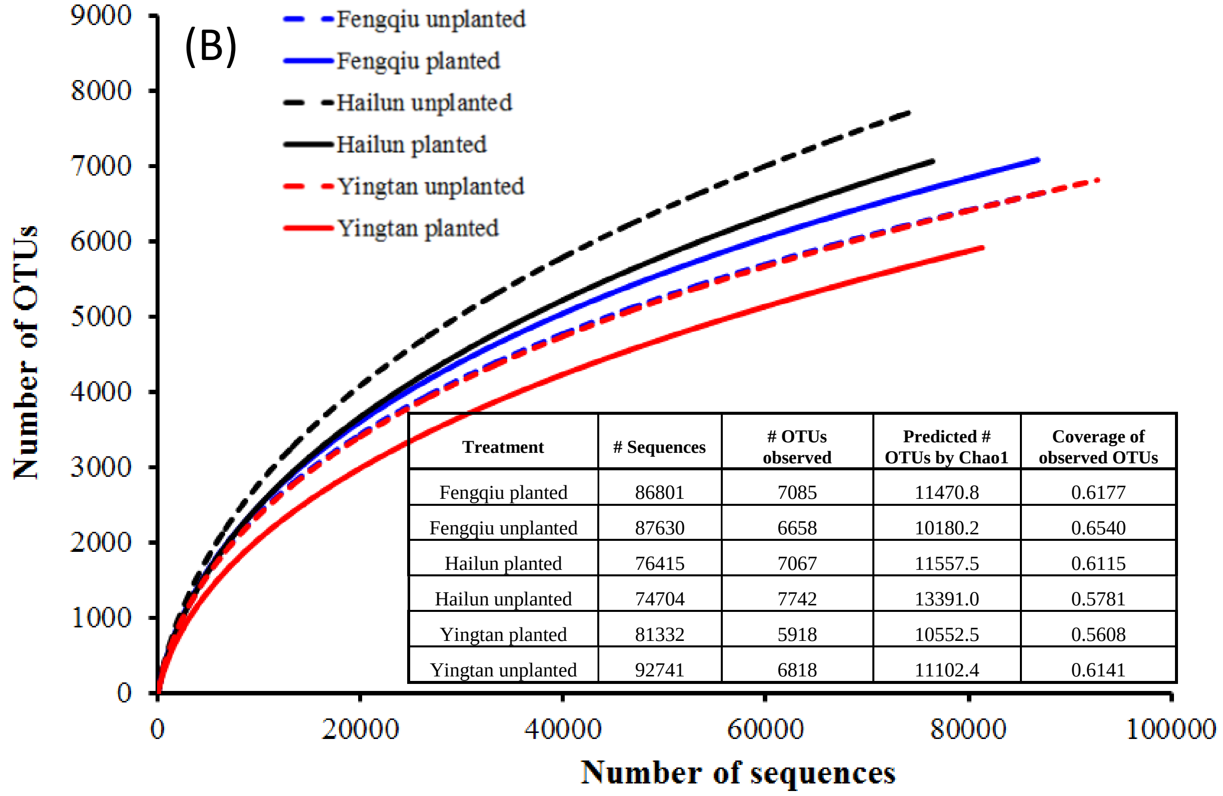

Treatment	# Sequences	# OTUs observed	Predicted # OTUs by Chao1	Coverage of observed OTUs
Fengqiu planted	86801	7085	11470.8	0.6177
Fengqiu unplanted	87630	6658	10180.2	0.6540
Hailun planted	76415	7067	11557.5	0.6115
Hailun unplanted	74704	7742	13391.0	0.5781
Yingtan planted	81332	5918	10552.5	0.5608
Yingtan unplanted	92741	6818	11102.4	0.6141

S 1B Fig. Rarefaction analysis based on treatments for experiment II.
