# Supplementary material for: Evaluation of the reproducibility of amplicon sequencing with Illumina MiSeq platform
Source: PLoS One. 2017 Apr 28;12(4):e0176716. doi: 10.1371/journal.pone.0176716 (PMC5409056; doi:10.1371/journal.pone.0176716)
Supplement: S2 Fig — (A) Between two technical replicates, singletons not removed; (B) between two technical replicates, singletons removed; (C) among three technical replicates, singletons not removed; (D) among three technical replicates, singletons removed. (PDF) [file pone.0176716.s002.pdf]

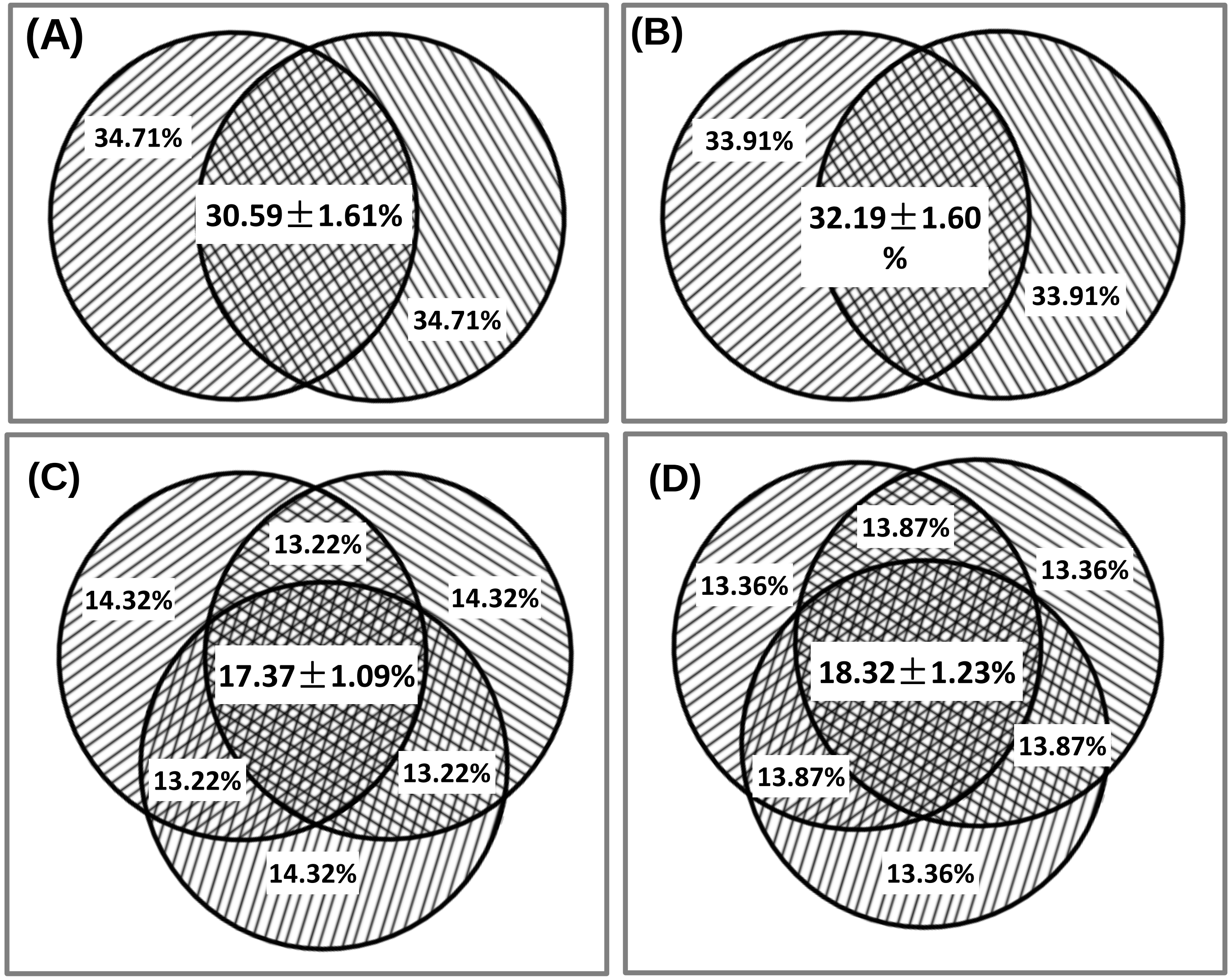

S2 Fig. OTU overlaps between/among technical replicates for experiment II. (A) Between two technical replicates, singletons not removed; (B) between two technical replicates, singletons removed; (C) among three technical replicates, singletons not removed; (D) among three technical replicates, singletons removed.
