# Supplementary material for: Evaluation of the reproducibility of amplicon sequencing with Illumina MiSeq platform
Source: PLoS One. 2017 Apr 28;12(4):e0176716. doi: 10.1371/journal.pone.0176716 (PMC5409056; doi:10.1371/journal.pone.0176716)
Supplement: S3 Fig — At a sequencing depth of about 30,000 reads, overlap of both two and three replicates were reaching a plateau no matter singletons were removed or not. (PDF) [file pone.0176716.s003.pdf]

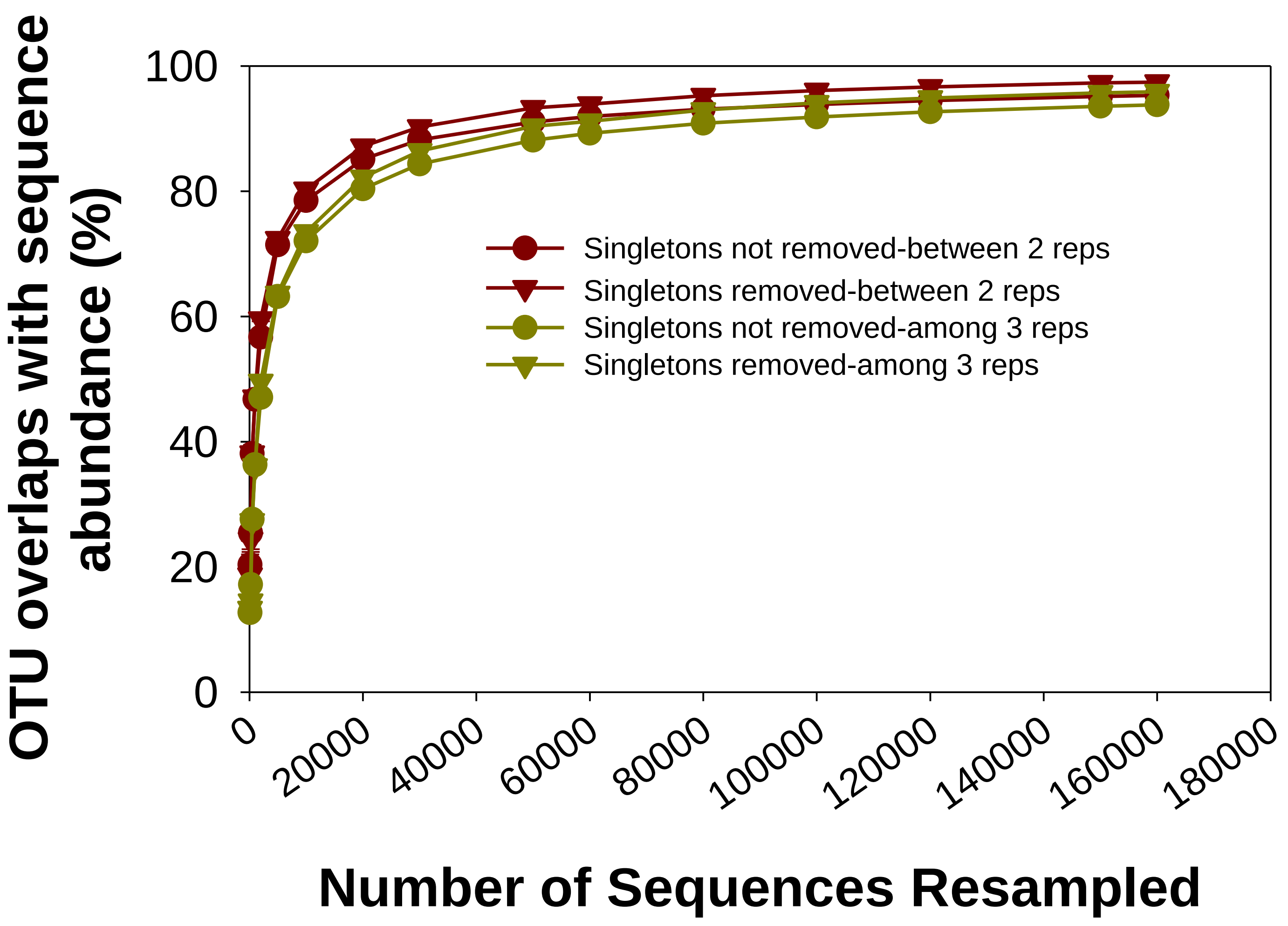

S3 Fig. Sequence abundance weighted OTU Overlaps generated with using Uclust between/among technical replicates at different sequencing depth. At a sequencing depth of about 30,000 reads, overlaps of both two and three replicates were reaching a plateau no matter singletons were removed or not.
