# Supplementary material for: Evaluation of the reproducibility of amplicon sequencing with Illumina MiSeq platform
Source: PLoS One. 2017 Apr 28;12(4):e0176716. doi: 10.1371/journal.pone.0176716 (PMC5409056; doi:10.1371/journal.pone.0176716)
Supplement: S5 Fig — Samples were from three experiment locations (H, Hailun; F, Fengqiu; Y, Yingtan) with two treatments at each location: planted (P) and unplanted (C, control), each treatment with three field replicates. Each soil was tagged three times as technical replicates. For experiment I, the three technical replicates of each soil were sequenced in the same MiSeq run, and for experiment II, the three technical replicates of each soil were sequenced in three different MiSeq runs. (PDF) [file pone.0176716.s005.pdf]

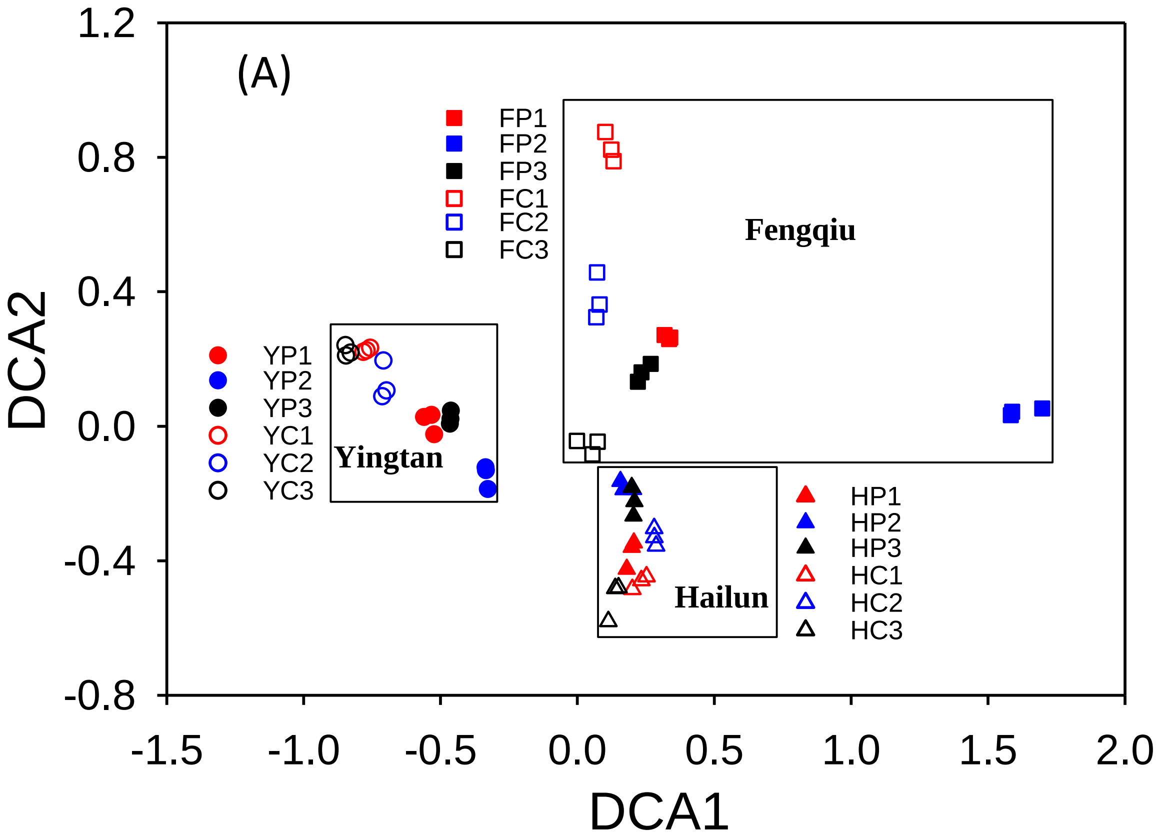


**S5A Fig. Detrented corresponding analysis of the microbial communities for experiment I, with singletons removed.** Samples were from three experiment locations (H, Hailun; F, Fengqiu; Y, Yingtan) with two treatments at each location: planted (P) and unplanted (C, control), each treatment with three field replicates. Each soil was tagged three times as technical replicates. The three technical replicates of each soil were sequenced in the same MiSeq run. Singletons were removed.

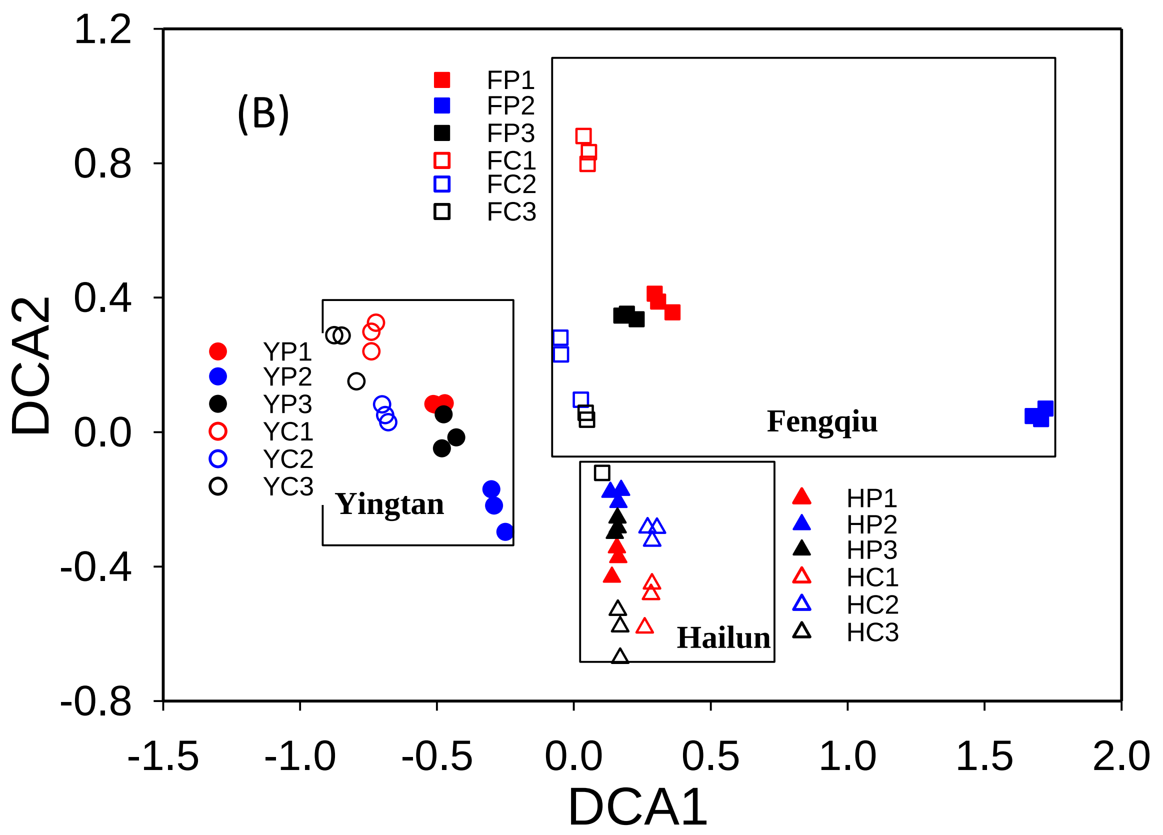


**S5B Fig. Detrented corresponding analysis of the microbial communities for experiment II, with singletons not removed.** Samples were from three experiment locations (H, Hailun; F, Fengqiu; Y, Yingtan) with two treatments at each location: planted (P) and unplanted (C, control), each treatment with three field replicates. Each soil was tagged three times as technical replicates. The three technical replicates of each soil were sequenced in three different MiSeq runs. Singletons were not removed.

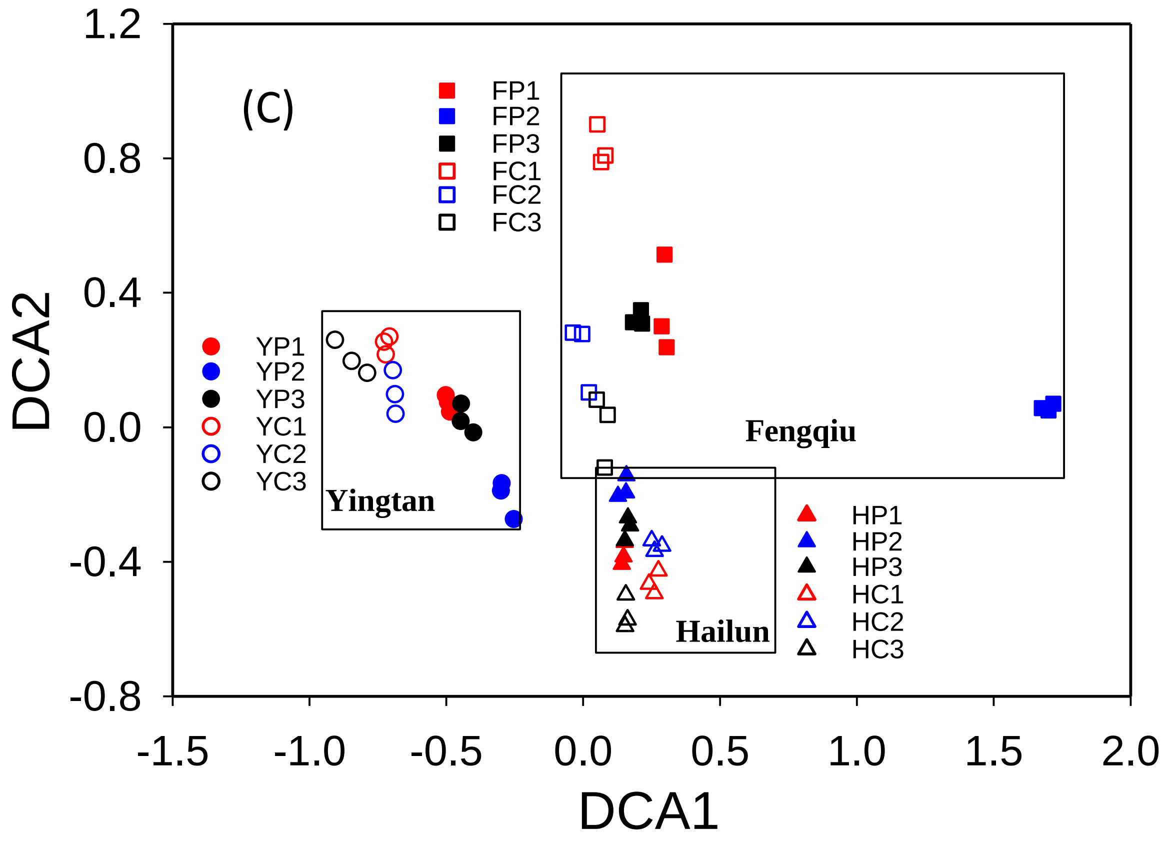


**S5C Fig. Detrented corresponding analysis of the microbial communities for experiment II, with singletons removed.** Samples were from three experiment locations (H, Hailun; F, Fengqiu; Y, Yingtan) with two treatments at each location: planted (P) and unplanted (C, control), each treatment with three field replicates. Each soil was tagged three times as technical replicates. The three technical replicates of each soil were sequenced in three different MiSeq runs. Singletons were removed.
