# Supplementary material for: Evaluation of the reproducibility of amplicon sequencing with Illumina MiSeq platform
Source: PLoS One. 2017 Apr 28;12(4):e0176716. doi: 10.1371/journal.pone.0176716 (PMC5409056; doi:10.1371/journal.pone.0176716)
Supplement: S2 Table — (PDF) [file pone.0176716.s007.pdf]

S2 Table. Sample (tagged PCR libraries) arrangement in MiSeq runs and sequencing parameters

Experiment		Number of Samples		Cluster density (k/mm^2)	Clusters passed Filter (%)	Reads Q=>30(%)	PhiX (%)
		This study	Others [c]				
**I [a]**	Run 1	54	16	563	93.7	80.1	45.9
**II [b]**	Run 1	18	77	453	95.5	85.5	47.3
	Run 2	18	75	531	93.8	83.9	52.5
	Run 3	18	77	580	93.1	82.3	54.2

[a] Experiment I: The three technical replicates (tagged PCR libraries) of each soil samples, and the total 54 tagged PCR libraries of all the 18 soil samples in this study were all sequenced in one MiSeq run.

[b] Experiment II: The three technical replicates (tagged PCR libraries) of each soil sample were arranged in three different MiSeq runs, in each of the three MiSeq runs there were 18 tagged PCR libraries, one from each of the 18 soil samples.

[c] Others: Other tagged PCR libraries from other experiments which pooled and sequenced with the tagged PCR libraries from this study.
