# Supplementary material for: Evaluation of the reproducibility of amplicon sequencing with Illumina MiSeq platform
Source: PLoS One. 2017 Apr 28;12(4):e0176716. doi: 10.1371/journal.pone.0176716 (PMC5409056; doi:10.1371/journal.pone.0176716)
Supplement: S3 Table — (PDF) [file pone.0176716.s008.pdf]

S3 Table. Summary of sequencing statistics for experiment I

Tagged PCR Library	Total raw sequences	Effective sequences for analysis	# of OTUs (97% cutoff)	# of OTUs after removing singletons	Predicted # OTUs by Chao1	
					Number	Coverage of observed OTUs
**FP11**	13385	12500	2976	2811	5745.5	0.518
**FP12**	14578	13702	3049	2888	5784.4	0.527
**FP13**	14949	13979	2957	2801	5373.1	0.550
**FP21**	16745	15412	2350	2172	5297.2	0.444
**FP22**	15183	14164	2166	2039	4680.8	0.463
**FP23**	17800	16667	2413	2248	5085.8	0.474
**FP31**	16177	15523	2828	2724	5191.2	0.545
**FP32**	15115	14443	2795	2688	5174.5	0.540
**FP33**	18184	16935	3187	3001	6172.2	0.516
**FC11**	14100	13464	2733	2583	5304.7	0.515
**FC12**	12335	11743	2586	2460	5007.8	0.516
**FC13**	12804	12211	2695	2563	5075.8	0.531
**FC21**	16123	15353	2778	2684	4952.4	0.561
**FC22**	14749	14140	2680	2574	4994.0	0.537
**FC23**	17292	16118	2996	2847	5694.8	0.526
**FC31**	18116	15853	3199	2787	7434.8	0.430
**FC32**	16418	15109	2626	2459	5665.9	0.463
**FC33**	20393	18535	2932	2720	6103.2	0.480
**HP11**	16586	15376	2990	2809	6520.5	0.459
**HP12**	16651	15197	2942	2734	6360.4	0.463
**HP13**	16385	14878	2818	2597	5995.3	0.470
**HP21**	11295	10323	2435	2288	5621.5	0.433
**HP22**	17105	15320	3350	3049	7380.8	0.454
**HP23**	14833	13393	3168	2923	6795.1	0.466
**HP31**	15772	14558	3131	2928	6501.7	0.482
**HP32**	15829	14447	3164	2903	6728.4	0.470
**HP33**	16175	14853	3260	3031	6494.9	0.502
**HC11**	17631	15887	3838	3454	8081.2	0.475
**HC12**	17940	16420	3738	3407	7834.4	0.477
**HC13**	15959	14017	3469	3114	7819.9	0.444
**HC21**	15416	14400	3518	3285	6975.3	0.504
**HC22**	17906	16385	3732	3391	7481.5	0.499
**HC23**	16604	14954	3657	3270	7202.5	0.508
**HC31**	17668	16163	3212	2955	6615.5	0.486

**HC32**	18773	16864	3384	3065	7410.0	0.457
**FC33**	18079	16283	3390	3083	7177.5	0.472
**YP11**	18068	16253	2991	2717	6569.5	0.455
**YP12**	20179	18156	3287	2979	6941.9	0.474
**YP13**	20798	18684	3363	3036	7163.2	0.469
**YP21**	20007	17986	2741	2495	6127.8	0.447
**YP22**	13615	12448	2140	2022	4366.0	0.490
**YP23**	16997	15373	2480	2293	5620.8	0.441
**YP31**	17605	15961	2950	2729	5797.9	0.509
**YP32**	20636	18648	3389	3065	7275.9	0.466
**YP33**	15909	14370	2954	2684	6282.3	0.470
**YC11**	17456	15998	3278	2977	7178.5	0.457
**YC12**	17694	16308	3204	2940	6814.9	0.470
**YC13**	16666	15090	3241	2947	6951.7	0.466
**YC21**	20574	18623	3574	3195	7163.3	0.499
**YC22**	20078	18161	3495	3122	7569.0	0.462
**YC23**	16015	14739	3150	2868	6796.1	0.463
**YC31**	18336	16585	3490	3096	7612.3	0.458
**YC32**	19455	17746	3469	3096	7278.6	0.477
**YC33**	13442	12131	2922	2622	6672.6	0.438
Average	16751.54	15348.69	3060.37	2818.85	6368.8	0.483
Stdv	2188.19	1862.07	398.14	329.56	941.7	0.032
Subtotal	904583	828829	27774	14732	52502	0.529
