# Supplementary material for: Evaluation of the reproducibility of amplicon sequencing with Illumina MiSeq platform
Source: PLoS One. 2017 Apr 28;12(4):e0176716. doi: 10.1371/journal.pone.0176716 (PMC5409056; doi:10.1371/journal.pone.0176716)
Supplement: S4 Table — (PDF) [file pone.0176716.s009.pdf]

S4 Table. Summary of sequencing statistics for experiment II

Tagged PCR Library	MiSeq Run	Total raw sequences	Effective sequences for analysis	# of OTUs (97% cutoff)	# of OTUs after removing singletons	Predicted # OTUs by Chao1	
						Number	Coverage of observed OTUs
**FP11**	1	11390	10684	2809	2630	5277.2	0.532
**FP12**	2	11419	10435	2689	2542	5111.3	0.526
**FP13**	3	8661	7911	2307	2192	4290.4	0.538
**FP21**	1	13322	11849	2056	1939	3960.6	0.519
**FP22**	2	11705	10505	1793	1709	3681.3	0.487
**FP23**	3	7666	6844	1342	1289	3149.1	0.426
**FP31**	1	15878	15277	3216	3023	5616.5	0.573
**FP32**	2	9545	9166	2311	2209	4199.7	0.550
**FP33**	3	4161	4130	1447	1413	2868.8	0.504
**FC11**	1	16058	14980	3127	2921	5297.8	0.590
**FC12**	2	8061	7654	2111	2008	4226.8	0.499
**FC13**	3	6189	5868	1822	1742	3579.2	0.509
**FC21**	1	13245	12262	2658	2527	4956.6	0.536
**FC22**	2	6185	5725	1554	1492	3288.4	0.473
**FC23**	3	7265	6713	1764	1684	3516.9	0.502
**FC31**	1	14498	14189	2648	2543	4865.4	0.544
**FC32**	2	10671	10399	2100	2046	3927.8	0.535
**FC33**	3	10691	9840	1977	1898	4109.3	0.481
**HP11**	1	14459	12821	2844	2611	6174.8	0.461
**HP12**	2	10874	10084	2350	2219	4867.7	0.483
**HP13**	3	7983	7444	2001	1912	4144.1	0.483
**HP21**	1	10051	9240	2486	2347	5013.0	0.496
**HP22**	2	5102	4513	1500	1419	3909.5	0.384
**HP23**	3	5940	5495	1661	1586	3619.6	0.459
**HP31**	1	11527	10649	2717	2534	5417.4	0.502
**HP32**	2	7512	6963	1941	1837	3896.5	0.498
**HP33**	3	10156	9206	2222	2099	4696.2	0.473
**HC11**	1	13020	11210	3090	2804	6231.3	0.496
**HC12**	2	9956	9027	2589	2426	5059.6	0.512
**HC13**	3	7739	7066	2266	2133	4725.3	0.480
**HC21**	1	9422	8664	2672	2456	5382.8	0.496
**HC22**	2	5966	5385	1900	1781	4064.7	0.467

**HC23**	3	5381	4973	1771	1671	3918.0	0.452
**HC31**	1	11576	10562	2722	2531	5552.7	0.490
**HC32**	2	8447	7643	2103	1992	5099.0	0.412
**FC33**	3	11209	10174	2446	2279	5184.7	0.472
**YP11**	1	13578	12137	2416	2240	4633.6	0.521
**YP12**	2	9491	8926	1904	1825	3449.0	0.552
**YP13**	3	7461	7042	1654	1578	3402.6	0.486
**YP21**	1	11694	10474	1806	1686	3756.3	0.481
**YP22**	2	9830	8996	1639	1582	3315.6	0.494
**YP23**	3	6940	6326	1238	1187	2920.8	0.424
**YP31**	1	11495	10428	2276	1705	4540.1	0.501
**YP32**	2	8566	7773	1821	2088	3786.1	0.481
**YP33**	3	10221	9230	1911	1817	4005.3	0.477
**YC11**	1	13361	11958	2637	2450	4732.6	0.557
**YC12**	2	6601	6181	1707	1644	3570.8	0.478
**YC13**	3	5118	4759	1438	1393	2880.7	0.499
**YC21**	1	17606	16059	3182	2925	5592.9	0.569
**YC22**	2	12231	10973	2339	2203	4461.9	0.524
**YC23**	3	8679	7947	1955	1866	3820.6	0.512
**YC31**	1	16731	15395	3046	2806	5449.5	0.559
**YC32**	2	12406	11423	2408	2271	4480.8	0.537
**YC33**	3	8965	8046	1909	1811	3801.0	0.502
Average		10072.3	9252.3	2190.7	2065.2	4360.7	0.500
Stdv		3174.81	2912.1	507.7	456.3	846.7	0.040
Subtotal		543904	499623	17107	10330	28570	0.599
