# Supplementary material for: Evaluation of the reproducibility of amplicon sequencing with Illumina MiSeq platform
Source: PLoS One. 2017 Apr 28;12(4):e0176716. doi: 10.1371/journal.pone.0176716 (PMC5409056; doi:10.1371/journal.pone.0176716)
Supplement: S5 Table — (PDF) [file pone.0176716.s010.pdf]

S5 Table. OTU overlaps [a] between/among technical replicates for experiment I

Soil Sample	With singletons		Singletons removed	
	**Two tags**	**Three tags**	**Two tags**	**Three tags**
FP1	0.366±0.008	0.228	0.392±0.001	0.247
FP2	0.314±0.009	0.185	0.338±0.006	0.199
FP3	0.360±0.004	0.220	0.377±0.003	0.235
FC1	0.364±0.006	0.225	0.391±0.005	0.248
FC2	0.376±0.011	0.237	0.383±0.013	0.238
FC3	0.299±0.019	0.174	0.330±0.010	0.195
HP1	0.324±0.007	0.191	0.350±0.009	0.210
HP2	0.327±0.005	0.197	0.359±0.007	0.218
HP3	0.335±0.002	0.204	0.368±0.001	0.226
HC1	0.320±0.010	0.189	0.359±0.005	0.215
HC2	0.342±0.010	0.208	0.383±0.007	0.239
HC3	0.320±0.004	0.187	0.349±0.002	0.208
YP1	0.320±0.003	0.189	0.351±0.003	0.211
YP2	0.327±0.004	0.196	0.346±0.010	0.206
YP3	0.327±0.011	0.198	0.355±0.003	0.211
YC1	0.338±0.001	0.208	0.376±0.006	0.236
YC2	0.327±0.011	0.195	0.370±0.007	0.227
YC3	0.330±0.003	0.199	0.370±0.005	0.228
Average	**0.334±0.021**	**0.202±0.017**	**0.364±0.019**	**0.222±0.016**

[a] Before the OTU overlaps were calculated, each sample was rarefied at the level of 10323 sequences, the least sequence number of the 54 libraries.
