# Supplementary material for: Evaluation of the reproducibility of amplicon sequencing with Illumina MiSeq platform
Source: PLoS One. 2017 Apr 28;12(4):e0176716. doi: 10.1371/journal.pone.0176716 (PMC5409056; doi:10.1371/journal.pone.0176716)
Supplement: S6 Table — (PDF) [file pone.0176716.s011.pdf]

S6 Table. OTU overlaps between/among technical replicates for experiment II

Soil Sample	With singletons		Removing singletons	
	Two tags	Three tags	Two tags	Three tags
FP1	0.312±0.011	0.175	0.325±0.011	0.178
FP2	0.290±0.011	0.160	0.310±0.005	0.179
FP3	0.315±0.012	0.180	0.334±0.004	0.192
FC1	0.319±0.009	0.185	0.340±0.009	0.201
FC2	0.315±0.003	0.183	0.329±0.005	0.192
FC3	0.319±0.014	0.179	0.313±0.022	0.172
HP1	0.284±0.013	0.157	0.298±0.014	0.161
HP2	0.284±0.010	0.154	0.311±0.009	0.174
HP3	0.292±0.010	0.164	0.305±0.004	0.166
HC1	0.298±0.011	0.163	0.321±0.017	0.181
HC2	0.298±0.004	0.169	0.319±0.011	0.181
HC3	0.292±0.010	0.165	0.305±0.006	0.169
YP1	0.313±0.012	0.180	0.321±0.015	0.179
YP2	0.304±0.011	0.174	0.328±0.013	0.192
YP3	0.309±0.003	0.179	0.326±0.014	0.192
YC1	0.331±0.009	0.191	0.348±0.011	0.207
YC2	0.312±0.004	0.181	0.334±0.004	0.196
YC3	0.321±0.017	0.186	0.327±0.008	0.188
Average	**0.306±0.016**	**0.174±0.011**	**0.322±0.016**	**0.183±0.012**

[a] Before the OTU overlaps were calculated, each sample was rarefied at the level of 4130 sequences, the least sequence number of the 54 libraries.
