# Supplementary material for: Evaluation of the reproducibility of amplicon sequencing with Illumina MiSeq platform
Source: PLoS One. 2017 Apr 28;12(4):e0176716. doi: 10.1371/journal.pone.0176716 (PMC5409056; doi:10.1371/journal.pone.0176716)
Supplement: S7 Table — (PDF) [file pone.0176716.s012.pdf]

S7 Table. Sequence abundance weighted OTU overlap between/among technical replicates for experiment I

Soil Sample	With singletons		Removing singletons	
	Two tags	Three tags	Two tags	Three tags
FP1	0.853 ±0.005	0.804	0.864 ±0.004	0.813
FP2	0.888 ±0.003	0.855	0.898 ±0.002	0.865
FP3	0.864 ±0.002	0.817	0.870 ±0.003	0.824
FC1	0.861 ±0.004	0.816	0.872 ±0.004	0.829
FC2	0.873 ±0.003	0.831	0.877 ±0.003	0.832
FC3	0.859 ±0.015	0.821	0.871 ±0.011	0.832
HP1	0.857 ±0.002	0.813	0.869 ±0.004	0.825
HP2	0.844 ±0.005	0.798	0.859 ±0.005	0.812
HP3	0.848 ±0.001	0.803	0.862 ±0.003	0.812
HC1	0.824 ±0.005	0.772	0.844 ±0.003	0.788
HC2	0.831 ±0.002	0.779	0.850 ±0.004	0.796
HC3	0.848 ±0.000	0.801	0.862 ±0.001	0.815
YP1	0.861 ±0.003	0.820	0.873 ±0.002	0.831
YP2	0.886 ±0.001	0.852	0.891 ±0.002	0.853
YP3	0.859 ±0.006	0.819	0.870 ±0.002	0.824
YC1	0.854 ±0.001	0.811	0.869 ±0.004	0.826
YC2	0.849 ±0.002	0.804	0.869 ±0.002	0.822
YC3	0.847 ±0.002	0.801	0.864 ±0.002	0.816
Average	**0.856 ±0.016**	**0.812 ±0.021**	**0.869 ±0.013**	**0.823 ±0.018**
