# Supplementary material for: Evaluation of the reproducibility of amplicon sequencing with Illumina MiSeq platform
Source: PLoS One. 2017 Apr 28;12(4):e0176716. doi: 10.1371/journal.pone.0176716 (PMC5409056; doi:10.1371/journal.pone.0176716)
Supplement: S8 Table — (PDF) [file pone.0176716.s013.pdf]

S8 Table. Sequence abundance weighted OTU overlap between/among technical replicates for experiment II

Soil Sample	With singletons		Removing singletons	
	Two tags	Three tags	Two tags	Three tags
FP1	0.750 ±0.012	0.670	0.753 ±0.010	0.661
FP2	0.837 ±0.010	0.788	0.845 ±0.005	0.798
FP3	0.764 ±0.012	0.688	0.775 ±0.004	0.697
FC1	0.765 ±0.012	0.692	0.775 ±0.009	0.700
FC2	0.778 ±0.015	0.715	0.787 ±0.011	0.721
FC3	0.805 ±0.010	0.738	0.804 ±0.010	0.736
HP1	0.773 ±0.005	0.709	0.779 ±0.008	0.707
HP2	0.757 ±0.010	0.688	0.772 ±0.009	0.701
HP3	0.768 ±0.009	0.701	0.776 ±0.006	0.701
HC1	0.741 ±0.010	0.657	0.759 ±0.009	0.678
HC2	0.738 ±0.005	0.661	0.752 ±0.007	0.673
HC3	0.763 ±0.009	0.696	0.774 ±0.014	0.700
YP1	0.809 ±0.009	0.753	0.813 ±0.012	0.748
YP2	0.843 ±0.009	0.797	0.855 ±0.009	0.809
YP3	0.802 ±0.007	0.743	0.816 ±0.009	0.759
YC1	0.790 ±0.013	0.719	0.804 ±0.013	0.736
YC2	0.788 ±0.013	0.725	0.797 ±0.009	0.732
YC3	0.793 ±0.011	0.727	0.796 ±0.007	0.727
**Average**	**0.781 ±0.031**	**0.715 ±0.039**	**0.791 ±0.029**	**0.721 ±0.040**
