# Supplementary material for: Evaluation of the reproducibility of amplicon sequencing with Illumina MiSeq platform
Source: PLoS One. 2017 Apr 28;12(4):e0176716. doi: 10.1371/journal.pone.0176716 (PMC5409056; doi:10.1371/journal.pone.0176716)
Supplement: S9 Table — (PDF) [file pone.0176716.s014.pdf]

S9 Table. Effect of removing unique OTUs after sequence resampling on OTU overlaps between/among technical replicates [a]

Types of unique OTUs removed	OTUs number [b]	Two tags	Three tags
No OTU removed	14258	0.3637±0.0186	0.2221±0.0162
with **<=1** sequences removed	12324	0.3716±0.0187	0.2280±0.0164
with **<=2** sequences removed	12122	0.3725±0.0189	0.2287±0.0166
with **<=3** sequences removed	12108	0.3726±0.0188	0.2287±0.0166
with **<=4** sequences removed	12107	0.3726±0.0188	0.2287±0.0165
with **all** unique OTUs removed	12107	0.3726±0.0188	0.2287±0.0165

[a] OTUs were generated using Uclust at 97% similarity, and the data in this table is for the experiment I. Singletons were removed before sequence resampling.

[b] Before resampling, the OTUs number from OTU tables with and without singletons were 27774 and 14732, respectively. The OTUs numbers in the above table excluded the OTUs without any sequence which were resulted from resampling.
