# Supplementary material for: Evaluation of the reproducibility of amplicon sequencing with Illumina MiSeq platform
Source: PLoS One. 2017 Apr 28;12(4):e0176716. doi: 10.1371/journal.pone.0176716 (PMC5409056; doi:10.1371/journal.pone.0176716)
Supplement: S10 Table — (PDF) [file pone.0176716.s015.pdf]

S10 Table. OTU overlaps between/among technical replicates at different sequencing depth [b]

**# of Sequences Resampled [c]**	**With singletons**				**Without singletons**		
	**# of Total OTU**	**OTUs overlap**			**# of Total OTU**	**OTUs overlap**	
		Between	Among			Between	Among
**160000**	24745	**0.4391**	**0.3045**		14399	**0.6650**	**0.5257**
**150000**	24088	**0.4386**	**0.3039**		14362	**0.6553**	**0.5150**
**120000**	21862	**0.4331**	**0.2970**		14018	**0.6130**	**0.4685**
**100000**	20129	**0.4263**	**0.2902**		13647	**0.5875**	**0.4413**
**80000**	18158	**0.4222**	**0.2862**		13018	**0.5544**	**0.4054**
**60000**	15813	**0.4163**	**0.2793**		12141	**0.5173**	**0.3678**
**50000**	14615	**0.4073**	**0.2707**		11366	**0.5040**	**0.3554**
**30000**	11341	**0.3896**	**0.2529**		9510	**0.4541**	**0.3068**
**20000**	9328	**0.3668**	**0.2311**		8087	**0.4161**	**0.2686**
**10000**	6531	**0.3199**	**0.1877**		5953	**0.3528**	**0.2110**
**5000**	4309	**0.2873**	**0.1592**		4098	**0.2985**	**0.1635**
**2000**	2501	**0.2067**	**0.1004**		2339	**0.2262**	**0.1116**
**1000**	1540	**0.1641**	**0.0708**		1514	**0.1706**	**0.0740**
**500**	892	**0.1281**	**0.0493**		899	**0.1378**	**0.0534**
**200**	437	**0.0813**	**0.0275**		429	**0.0863**	**0.0256**
**100**	230	**0.0688**	**0.0261**		239	**0.0527**	**0.0167**

[a] Jaccard overlap, not considering sequence abundances of OTUs.

[b] One soil sample were tagged three times and deep sequenced. More than 160,000 sequences were obtained for each tagged PCR library.

[c] Sequence resampling was done after OTUs were generated.
