# Supplementary material for: Evaluation of the reproducibility of amplicon sequencing with Illumina MiSeq platform
Source: PLoS One. 2017 Apr 28;12(4):e0176716. doi: 10.1371/journal.pone.0176716 (PMC5409056; doi:10.1371/journal.pone.0176716)
Supplement: S11 Table — (PDF) [file pone.0176716.s016.pdf]

S11 Table. Sequence abundance weighted OTU overlap between/among technical replicates at different sequence depth

**# of Sequences resampled**	**With singletons**			**Without singletons**		
	**# of OTU**	**Sequences overlap**		**OTU#**	**Sequences overlap**	
		Between two tags	Among three tags		Between two tags	Among three tags
160000	24745	**0.9530**	**0.9379**	14399	**0.9742**	**0.9587**
150000	24088	**0.9513**	**0.9357**	14362	**0.9729**	**0.9570**
120000	21862	**0.9448**	**0.9267**	14018	**0.9665**	**0.9487**
100000	20129	**0.9385**	**0.9185**	13647	**0.9608**	**0.9409**
80000	18158	**0.9309**	**0.9085**	13018	**0.9525**	**0.9296**
60000	15813	**0.9192**	**0.8924**	12141	**0.9391**	**0.9117**
50000	14615	**0.9105**	**0.8814**	11366	**0.9329**	**0.9037**
30000	11341	**0.8819**	**0.8433**	9510	**0.9025**	**0.8640**
20000	9328	**0.8511**	**0.8035**	8087	**0.8716**	**0.8218**
10000	6531	**0.7849**	**0.7203**	5953	**0.8027**	**0.7348**
5000	4309	**0.7140**	**0.6317**	4098	**0.7238**	**0.6363**
2000	2501	**0.5668**	**0.4705**	2339	**0.5957**	**0.4960**
1000	1540	**0.4672**	**0.3630**	1514	**0.4708**	**0.3610**
500	892	**0.3807**	**0.2760**	899	**0.3817**	**0.2733**
200	437	**0.2542**	**0.1717**	429	**0.2433**	**0.1450**
100	230	**0.2033**	**0.1267**	239	**0.1867**	**0.1333**
