# Supplementary material for: Evaluation of the reproducibility of amplicon sequencing with Illumina MiSeq platform
Source: PLoS One. 2017 Apr 28;12(4):e0176716. doi: 10.1371/journal.pone.0176716 (PMC5409056; doi:10.1371/journal.pone.0176716)
Supplement: S12 Table — (PDF) [file pone.0176716.s017.pdf]

S12 Table. OTU overlaps between/among technical replicates at different sequencing depth with OTUs generated by UPARSE

**# of Sequences resampled**	**With singletons**				**Without singletons**		
	**# of Total OTU**	**OTUs overlap**			**# of Total OTU**	**OTUs overlap**	
		Between	Among			Between two	Among
**120000**	8309	**0.5786**	**0.4445**		4270	**0.7929**	**0.6988**
**100000**	7869	**0.5694**	**0.4337**		4242	**0.7605**	**0.6558**
**80000**	7342	**0.5572**	**0.4203**		4168	**0.7251**	**0.6101**
**60000**	6674	**0.5465**	**0.4093**		4055	**0.6855**	**0.5618**
**50000**	6336	**0.5273**	**0.3868**		3897	**0.6605**	**0.5309**
**30000**	5277	**0.5014**	**0.3597**		3543	**0.6010**	**0.4629**
**20000**	4516	**0.4815**	**0.3364**		3186	**0.5551**	**0.4109**
**10000**	3429	**0.4236**	**0.2794**		2529	**0.4914**	**0.3416**
**5000**	2513	**0.3840**	**0.2415**		1934	**0.4164**	**0.2725**
**2000**	1535	**0.2980**	**0.1668**		1231	**0.3483**	**0.2088**
**1000**	1029	**0.2442**	**0.1244**		900	**0.2689**	**0.1422**
**500**	692	**0.1885**	**0.0882**		575	**0.2258**	**0.1148**
**200**	333	**0.1445**	**0.0571**		328	**0.1510**	**0.0579**
**100**	193	**0.0784**	**0.0207**		197	**0.1127**	**0.0406**
