# Supplementary material for: Evaluation of the reproducibility of amplicon sequencing with Illumina MiSeq platform
Source: PLoS One. 2017 Apr 28;12(4):e0176716. doi: 10.1371/journal.pone.0176716 (PMC5409056; doi:10.1371/journal.pone.0176716)
Supplement: S13 Table — (PDF) [file pone.0176716.s018.pdf]

S13 Table. Three-way ANOVA [a] to assess alpha diversities [b] at different levels for experiment II.

	Df	Shannon measurement (H')				Number of OTUs				Pielou evenness (J)			
		With singletons		Singletons removed		With singletons		Singletons removed		With singletons		Singletons removed	
		F [c]	P [d]	F	P	F	P	F	P	F	P	F	P
Technical replicate	2	1.343	0.271	1.262	0.292	2.771	0.073	2.085	0.135	1.011	0.371	1.062	0.354
Location	2	6.389	0.003**	5.625	0.006**	12.702	4.E-05**	10.681	1.5E-04**	4.904	0.012*	4.36	0.018*
Treatment	1	14.116	4.7E-04**	12.674	0.001**	9.667	0.003**	8.506	0.005**	15.301	2.9E-04**	13.80	0.001**
Residuals	48												

[a] The model used for the three-way ANOVA: $(\alpha \text{ diversity})_{ijk}=(\text{technical replicate})_i+(\text{location})_j+(\text{treatment})_k+(\text{error})_{ijk}$.

[b] The Shannon entropy index, number of OTUs, and the Pielou evenness were used to measure the alpha diversity for each tagged PCR library.

[c] F-value

[d] P-value (>F)

* p<0.05

** p<0.01
