# Supplementary material for: Evaluation of the reproducibility of amplicon sequencing with Illumina MiSeq platform
Source: PLoS One. 2017 Apr 28;12(4):e0176716. doi: 10.1371/journal.pone.0176716 (PMC5409056; doi:10.1371/journal.pone.0176716)
Supplement: S14 Table — (PDF) [file pone.0176716.s019.pdf]

S14 Table. One-way ANOVA and Duncan grouping to assess β-diversity at different levels based on OTUs for experiment II.

	Data size [a]	With singletons				Singletons removed			
		Sorensen		Bray-Curtis		Sorensen		Bray-Curtis	
		β diversity [b]	Significance [c]	β diversity [b]	Significance [c]	β diversity [b]	Significance [c]	β diversity [b]	Significance [c]
At technical replicate level	54	0.531	d	0.361	d	0.513	e	0.354	d
At biological replicate level within runs	54	0.554	c	0.434	c	0.535	d	0.425	c
At biological replicate level among runs	108	0.558	c	0.442	c	0.541	d	0.436	c
Between treatments (Planted/ unplanted) within locations	243	0.588	b	0.489	b	0.572	c	0.482	b
Among planted across locations	243	0.646	a	0.561	a	0.630	b	0.556	a
Among unplanted across locations	243	0.651	a	0.568	a	0.639	a	0.564	a

[a] Data sizes (n) are the number of data points of the pairwise comparisons within the technical replicates, biological replicates, or treatments.

[b] We calculate two popular β-diversity dissimilarity measurements, Sorensen and Bray-Curtis, in which Sorensen dissimilarity is based on OTUs richness and Bray-Curtis dissimilarity takes OTUs abundance into account.

[c] Significance at [pr(>F)] <0.05, using Duncan grouping method. a, b, c, d and e represent the significance of the β-diversity differences between technical replicates, biological replicates, and the treatments. a marks the highest β-diversity, the one less than the highest but not significant is still marked with a, then the ones significantly lower than the highest is marked with b, c, d, or e.
