# Supplementary material for: Evaluation of the reproducibility of amplicon sequencing with Illumina MiSeq platform
Source: PLoS One. 2017 Apr 28;12(4):e0176716. doi: 10.1371/journal.pone.0176716 (PMC5409056; doi:10.1371/journal.pone.0176716)
Supplement: S15 Table — (PDF) [file pone.0176716.s020.pdf]

S15 Table. Comparison of dispersions between observed technical replicates and those between null replicates [a] by permutation test of multivariate homogeneity of groups dispersions (PERMDISP).

Sample [a]	Sorensen (unweighted)			Bray-Curtis (abundance-weighted)		
	Δd% [c]	F [d]	P [e]	Δd% [c]	F [d]	P [e]
FP1	31±2%	3688±5425	0.001	15±2%	108±41	0.001
FP2	31±3%	562±185	0.001	22±1%	67±12	0.001
FP3	31±1%	1614±930	0.001	18±2%	61±15	0.001
FC1	29±2%	409±87	0.001	16±1%	49±10	0.001
FC2	31±1%	151±61	0.001	20±1%	138±8	0.001
FC3	33±3%	317±86	0.001	35±2%	10±1	0.001
HC1	28±2%	696±391	0.001	17±1%	96±23	0.001
HC2	27±2%	358±179	0.001	17±2%	31±6	0.001
HC3	31±1%	752±585	0.001	17±2%	44±13	0.001
HP1	31±2%	245±89	0.001	20±2%	25±5	0.001
HP2	30±1%	355±142	0.001	24±2%	587±262	0.001
HP3	30±2%	1629±1040	0.001	16±2%	24±6	0.001
YC1	30±1%	488±230	0.001	13±1%	115±70	0.001
YC2	29±1%	378±195	0.001	17±1%	24±2	0.001
YC3	29±1%	581±456	0.001	13±1%	50±16	0.001
YP1	30±2%	606±461	0.001	16±0%	45±9	0.001
YP2	32±3%	254±48	0.001	18±1%	43±7	0.001
YP3	31±2%	1483±784	0.001	17±2%	196±45	0.001

[a] Each sample has three technical replicates;

[b] The null replicates are about to simulate random draw of individuals from the combined community of observed technical replicates;

[c] Δd% is the relative difference between dispersion of observed replicates and dispersion of null replicates. Δd% values are all larger than 12%;

[d] F values are presented as mean ± standard deviation of the comparisons between observed and 1000 null datasets;

[e] P values are all significant, indicating the observed technical replicates are significantly more different from each other than null expectation of random sampling.
